# Tissue-specific expression of *Ruby* in Mexican lime (*C. aurantifolia*) confers anthocyanin accumulation in fruit

**DOI:** 10.3389/fpls.2022.945738

**Published:** 2022-08-08

**Authors:** Roger Thilmony, Kasturi Dasgupta, Min Shao, Daren Harris, Jake Hartman, Leslie A. Harden, Ron Chan, James G. Thomson

**Affiliations:** ^1^Crop Improvement and Genetics, Western Regional Research Center, United States Department of Agriculture (USDA)-Agricultural Research Service (ARS), Albany, CA, United States; ^2^Citrus Research Board, Visalia, CA, United States; ^3^Produce Safety and Microbiology Research, Western Regional Research Center, United States Department of Agriculture (USDA)-Agricultural Research Service (ARS), Albany, CA, United States

**Keywords:** anthocyanin, citrus (citrus sinensis), *CsMybA1*, *Ruby*, fruit biotechnology, promoter

## Abstract

Tissue specific promoters are important tools for the precise genetic engineering of crop plants. Four fruit-preferential promoters were examined for their ability to confer a novel fruit trait in transgenic Mexican lime (*Citrus aurantifolia*). The *Ruby* transcription factor activates fruit anthocyanin accumulation within Moro blood orange and has been shown to function in activating anthocyanin accumulation in heterologous plant species. Although the *CitVO1, CitUNK, SlE8*, and *PamMybA* promoters were previously shown to confer strong fruit-preferential expression in transgenic tomato, they exhibited no detectable expression in transgenic Mexican lime trees. In contrast, the *CitWax* promoter exhibited high fruit-preferential expression of *Ruby*, conferring strong anthocyanin accumulation within the fruit juice sac tissue and moderate activity in floral/reproductive tissues. In some of the transgenic trees with high levels of flower and fruit anthocyanin accumulation, juvenile leaves also exhibited purple coloration, but the color disappeared as the leaves matured. We show that the *CitWax* promoter enables the expression of *Ruby* to produce anthocyanin colored fruit desired by consumers. The production of this antioxidant metabolite increases the fruits nutritional value and may provide added health benefits.

## Introduction

Biotechnology offers the potential to improve agricultural crop production and facilitate the development to design novel commodities for use as food, feed, or fuel. One avenue of biotechnological improvement is the use of gene expression control elements (promoters) to precisely control when, where and how the introduced genes/traits will be expressed. Although numerous promoters have been identified that confer constitutive, or inducible expression in transgenic plants, fewer organ- or tissue-specific promoters that confer expression within specific cell or tissue types have been identified, particularly in crop plants.

Fruit are important sources of nutrients, minerals, vitamins, and dietary fiber in the human diet, and as such, significant efforts have been made to breed for fruit with higher yield, better quality, and other desirable traits. Citrus is one of the most important fruit tree crops worldwide their fruit are considered healthy food because they are low in fat and rich in dietary fiber, vitamin C, vitamin B (thiamin, pyridoxine, niacin, riboflavin, pantothenic acid, and folate), vitamin A, carotenoids, flavonoids, and limonoids (Liu et al., 2012). Most citrus fruit have an orange or yellow color due to the presence of carotenoids, except blood orange fruit which exhibit a bright purple color due to the presence of anthocyanins. Anthocyanins are synthesized from flavonoid precursors through a complex expression anthocyanin biosynthetic enzymes and regulatory genes including WD-repeat proteins, and basic helix-loop-helix (bHLH) and MYB transcription factors (Holton and Cornish, 1995; Baudry et al., 2004) and provide pigmentation to many plant tissues. Anthocyanin-activating MYB transcription factors have been identified in many plant species including Arabidopsis, *Citrus sinensis*, *Prunus americana, Ipomoea batatas*, and *Vitis vinifera* (Dasgupta et al., 2017).

The *Citrus* genus has a limited number of species that express anthocyanins within their fruit. The trait can appear in the young shoots and floral tissues of many lemon cultivars (Fabroni et al., 2016), and in specific ‘blood orange’ cultivars like ‘Tarocco, ‘Moro,’ and ‘Sanguinello’ fruit with purple flesh and rind produced, but anthocyanin accumulation is typically not observed within the young shoots or flowers. The presence of pigments like anthocyanins is believed to protect juvenile leaves from light stress and insect predation (Gould et al., 2000; Steyn et al., 2002; Karageorgou and Manetas, 2006). The Moro cultivar was previously shown to carry a novel copia-like retrotransposon sequence inserted within the promoter region of a MYB transcription factor *Ruby* and shown to be responsible for the activation of anthocyanin production in blood orange fruit (Butelli et al., 2012). Environmental factors also play a significant role in production of the blood orange trait and it appears to require hot, dry days followed by cool nights. Due to these specific conditions the blood orange is widely cultivated in southern Italy and Sicily. Mondello et al. (2000), Lo Piero (2015), but when grown in the subtropical areas, these varieties do not reliably accumulate sufficient anthocyanins to develop the purple color (Lee, 2002). The presence of anthocyanin pigments in these specific cultivars makes them attractive to consumers. In addition, anthocyanins have been shown to provide several health benefits if consumed in sufficient quantities (Bridle and Timberlake, 1997; Lo Piero, 2015). Previous studies have shown that anthocyanins are strong free radical scavengers (Sanchez-Moreno, 2002; De Beer et al., 2003) and have powerful antioxidant capacities (Deighton et al., 2000; Lila, 2004; Legua et al., 2022). Eating foods with anthocyanins has been linked to the prevention of a number of human health issues including obesity and diabetes (Tsuda et al., 2003). In addition to those health benefits, anthocyanins can also act as bacteriostatic agents (Naz et al., 2007) and are widely used as a natural source of food colorants (Manach et al., 2004). The accumulation of anthocyanin within plant tissues has also been correlated with enhanced drought, salt, and cold tolerance, and it has been shown to protect against insect herbivory and pathogen attack, UV-B, photo inhibition, and the accumulation of reactive oxygen species (Gould et al., 2000; Xu et al., 2017).

The overexpression of these MYB regulators has also been shown to induce anthocyanin accumulation in an array plant species (Borevitz et al., 2000; Gonzali et al., 2009; Meng et al., 2014; Xu et al., 2017). However, a suitable bHLH partner is often required to achieve full functionality in heterologous systems (Takos et al., 2006; Espley et al., 2007; Xiang et al., 2015). The beneficial properties of anthocyanins have motivated numerous researchers to enhance their production in various plants through metabolic engineering (Dixon et al., 2013). The development of genetically modified citrus with the ability to overexpress anthocyanins would potentially increase the health benefits of these fruits and allow the cultivation of these trees in more diverse environments. In addition, the modified citrus plants could also be used as ornamental plants or as alternative sources of easily extracted anthocyanins for use in food coloring or the nutritional enhancement of other foods (Scordino et al., 2015).

Our lab previously investigated the ability of a series of *MybA* transcription factors from Arabidopsis, citrus, grape and plum to confer anthocyanin accumulation in transgenic tobacco plants. The citrus MybA transcription factor *Ruby* from the Moro blood orange was shown to confer anthocyanin accumulation, seen as a bright fuchsia color, in transgenic tobacco and citrus plants (Dasgupta et al., 2017). The *Ruby* and *VvmybA1* transcription factor genes under the control of the constitutive CaMV35S promoter were also expressed in transgenic Mexican lime. These transgenic trees exhibited anthocyanin accumulation in multiple different tissues including leaves, flowers and fruit and some of the transgenic events with intense pigmentation had a stunted growth pattern and curled leaves (Dutt et al., 2016). Similar results have been observed in other species following constitutive accumulation of anthocyanins in transgenic plants (Goldsbrough et al., 1996; Bradley et al., 1998; Stover et al., 2013). The leaves of the *Ruby* transgenic trees exhibited a mottled purple color, pink flowers and fruit, but were typically less strongly pigmented than the *VvmybA1* transgenic events (Dutt et al., 2016; Hijaz et al., 2018).

To produce colored citrus fruit without need for specific environmental conditions or causing an unfavorable metabolic load and stunting plant growth our lab characterized several candidate promoters from citrus and plum that were found to confer fruit preferential expression in tomato (Dasgupta et al., 2020). The highest levels of transgene expression/activity were detected in fruit tissues including the pericarp, placenta, locule, and columella. Some of the promoters also exhibited weak activity in various reproductive or vegetative tissues (Dasgupta et al., 2020). The *CitWax* promoter exhibited expression that was similar to those for the tomato fruit ripening-specific promoters *SlE8* and *SlPG* (Xu et al., 1996; Lau et al., 2009). The *CitUNK, CitVO1*, and *PamMybA* promoters exhibited the strongest tomato fruit expression, but they also demonstrated expression in reproductive and/or vegetative tissues.

We investigate the use of four novel promoters (*CitWax*, *CitUNK, CitVO1*, and *PamMybA*) and compared to the published *SlE8* promoter for tissue specific expression of *Ruby* in Mexican lime. Transgenic plants were generated, molecularly and phenotypically characterized, and grown in the greenhouse for fruit production. The accumulation of anthocyanins within the fruit and other parts of the tree were evaluated. Results from this research will be informative for engineering citrus trees with robust and reliable fruit specific expression without compromising overall plant health. Results from this research and how these molecular tools may be useful in engineering citrus fruit with improved traits and nutritional quality will be discussed.

## Results and discussion

### Generation and molecular characterization of the transgenic Mexican lime trees

**FIGURE 1:**
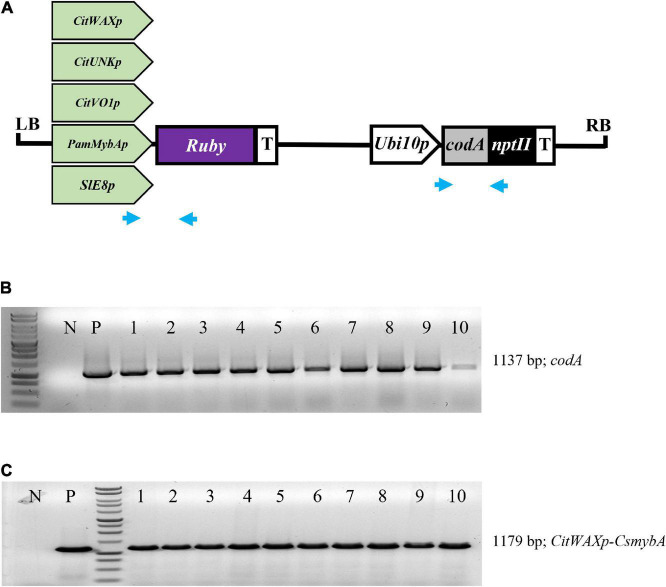
**(A)** Schematic representation of the promoter-*Ruby* construct T-DNAs. The *codA-npt*II selection marker gene is under the control of the Arabidopsis *Ubiquitin10* promoter (*Ubi10p*) and nopaline synthase transcriptional terminator (T). The test promoters from citrus (*CitWaxp*, *CitUNKp*, and *CitVO1p*) plum (*PamMybAp*) and tomato (*SlE8p*) are shown upstream of the *Ruby* (*CsmybA1*) gene. LB and RB designate the *Agrobacterium* left and right borders. Blue arrows indicate the approximate position of the *codA* and *Ruby* primers used for molecular validation. **(B)** PCR confirmation of the presence of *codA* transgene in 10 independent events. **(C)** PCR confirmation of the presence of the *CitWaxp-Ruby* sequence. P designates a sample with a plasmid DNA template. N indicates genomic DNA from wildtype Mexican lime. MW indicates molecular marker (Promega, 1 kb DNA ladder, G7541).

The *CitWax*, *CitVO1*, *CitUNK*, *PamMybA*, and control *SlE8* promoters were fused to the *Ruby* gene in a pCTAGII binary vector T-DNA (Figure 1A) and *Agrobacterium*-mediated transformation was used to generate transgenic Mexican lime trees. A total of 8 or more independent events were successfully transferred to soil and grown in a greenhouse for each promoter construct (Table 1). The transgenic events were each validated using PCR confirming the presence of the *codA* transgene and the junction between the promoter being tested and the *Ruby* sequence (Figures 1B,C and Supplementary Figure 1).

**TABLE 1:** Summary of the tree and fruit characterization.

Genotype/event	Copy #	Flower color	Fruit color	Anthocyanin	Brix	pH	SPAD value	Stomatal conductance
Mexican lime	0	−	−	0.00	7.31	2.05	40.4	223.5
CitVO1 1-1	6.6	−	−	0.00	6.90	2.22	28.8	152.7
CitVO1 1-5	1.1	−	−	0.00	7.54	2.02	35.3	161.1
CitVO1 1-6	1.1	−	−	0.00	8.19	2.04	52.2	275.7
CitVO1 1-11	1.3	−	−	0.00	7.90	2.06	44.5	181.2
CitVO1 1-16	1.1	−	−	0.00	8.48	2.04	51.8	133.7
CitVO1 1-22	1.1	−	−	0.00	7.09	2.13	47.6	154.8
CitVO1 1-24	1.1	−	−	0.00	7.45	2.07	56.5	201.0
CitVO1 1-25	1.1	−	−	0.00	7.45	2.14	66.5	405.8
CitVO1 1-26	1.0	−	−	0.00	8.56	2.09	49.3	191.4
CitUNK 4-3	2.9	−	−	0.00	8.35	2.13	39.3	288.5
CitUNK 4-5	1.0	−	−	0.00	8.57	2.05	47.8	147.9
CitUNK 4-37	4.1	−	−	0.00	6.84	2.22	43.1	424.0
CitUNK 4-38	1.1	−	−	0.00	8.74	2.14	42.9	165.2
CitUNK 4-40	1.1	−	−	0.00	7.95	2.02	41.6	413.0
CitUNK 4-42	1.1	−	−	0.00	7.29	2.16	40.2	405.0
CitUNK 4-44	2.4	−	−	0.00	7.45	2.08	55.3	326.5
CitUNK 4-47	1.1	−	−	0.00	8.59	2.17	57.9	534.6
CitUNK 4-56	1.4	−	−	0.00	8.26	2.04	53.3	463.2
CitUNK 4-57	1.0	−	−	0.00	8.00	2.09	40.9	179.7
CitUNK 4-70	0.9	−	−	0.00	8.84	2.07	32.8	190.0
CitWax 7-1	0.8	−	−	0.00	6.99	2.06	40.1	216.7
CitWax 7-2	2.1	+	+	0.05	7.06	2.14	43.0	167.7
CitWax 7-2A	1.0	+	+	0.08	8.04	2.47	54.7	230.0
CitWax 7-3	1.2	−	−	0.00	6.86	2.35	55.9	111.1
CitWax 7-5	1.1	+	+	0.00	7.67	2.13	49.2	176.4
CitWax 7-9	2.4	+	+	0.00	8.19	2.00	34.4	171.7
CitWax 7-10	2.2	+	+	0.19	7.83	2.04	48.0	152.9
CitWax 7-15	4.1	+	+	0.10	7.33	2.32	43.7	157.3
CitWax 7-16	4.0	+	+	0.24	7.24	2.12	47.8	363.0
CitWax 7-18	7.0	+	+	0.21	7.55	2.19	41.2	212.4
CitWax 7-19	4.0	+	+	0.30	6.56	2.23	48.1	236.2
CitWax 7-23	1.6	−	−	0.00	7.91	2.18	58.4	161.7
CitWax 7-24	6.3	+	+	0.66	7.93	2.13	27.7	261.7
CitWax 7-25	1.1	+	−	0.00	8.00	2.26	42.2	348.1
CitWax 9-1	1.1	+	+	0.21	6.90	2.32	54.7	185.2
CitWax 9-3	1.2	+	±	0.03	7.06	2.07	52.8	269.6
CitWax 9-6	1.1	+	+	0.04	7.95	2.08	50.1	157.2
CitWax 9-8	1.0	+	±	0.00	7.55	2.03	35.1	203.2
CitWax 9-8A	5.9	+	+	0.15	7.13	2.11	57.3	225.3
CitWax 9-9	6.5	+	+	0.41	5.66	2.15	57.1	310.0
CitWax 9-10	1.2	+	+	0.11	6.94	2.22	40.5	220.7
CitWax 9-12	6.6	+	+	0.30	7.14	2.24	55.6	302.0
CitWax 9-12A	2.3	+	+	0.10	7.62	2.15	46.5	160.6
CitWax 9-14	4.0	+	+	0.14	8.45	2.00	32.2	272.0
CitWax 9-15	6.1	+	+	0.42	6.89	1.97	45.3	253.3
CitWax 9-23	6.0	+	+	0.18	8.08	2.13	51.4	240.0
CitWax 9-24	7.5	+	+	0.30	7.79	2.01	48.7	452.8
CitWax 9-27	6.9	+	+	0.31	7.78	2.07	57.6	155.7
CitWax 9-28	8.1	+	+	0.28	7.77	2.05	45.7	199.7
CitWax 9-31	2.3	+	±	0.00	7.75	2.14	56.6	188.5
CitWax 9-36	5.2	+	+	0.32	7.21	2.09	52.4	135.4
CitWax 9-37	8.7	+	+	0.45	7.44	2.01	43.2	195.2
CitWax 9-38	2.2	+	+	0.08	7.71	2.23	47.2	162.2
CitWax 9-40	2.2	+	+	0.04	7.44	2.28	54.2	253.2
CitWax 9-41	2.8	+	+	0.06	7.47	2.23	32.3	185.5
CitWax 9-43	2.2	+	+	0.00	8.28	1.94	39.8	194.2
CitWax 9-100	4.2	+	+	0.12	8.56	2.04	ND	ND
PamMybA 5-2	3.6	−	−	0.00	7.01	2.15	30.1	160.7
PamMybA 5-3A	1.1	−	−	0.00	7.49	2.19	39.2	166.8
PamMybA 5-4	1.1	−	−	0.00	7.46	2.12	38.8	326.9
PamMybA 5-5	1.8	−	−	0.00	7.10	2.18	36.9	212.6
PamMybA 5-9	1.2	−	−	0.00	8.07	1.97	28.6	234.8
PamMybA 5-11	1.1	−	−	0.00	6.95	2.14	48.9	205.5
PamMybA 5-14	1.2	−	−	0.00	8.07	2.09	42.0	160.2
PamMybA 5-18	1.2	−	−	0.00	8.45	2.20	43.5	163.8
SlE8 10-2	1.5	−	−	0.00	7.95	2.07	48.5	254.6
SlE8 10-3	0.6	−	−	0.00	8.13	2.09	43.7	157.3
SlE8 10-9	3.8	−	−	0.00	7.58	2.03	42.4	170.3
SlE8 10-10	ND	−	−	0.00	7.65	2.05	51.0	208.7
SlE8 10-11	1.8	−	−	0.00	7.95	2.07	54.6	474.6
SlE8 10-13	2.3	−	−	0.00	7.57	1.94	29.5	205.7
SlE8 10-14	4.5	−	−	0.00	7.28	2.09	29.8	324.8
SlE8 10-18	1.5	−	−	0.00	8.08	2.09	28.4	276.6
Blood Orange	0.0	−	+	0.82	11.74	3.35	66.5	207.9

The measured *nptII* transgene copy number is shown (Copy #). A plus sign (+) indicates visible anthocyanin accumulation within the stigma and/or other reproductive tissues (Flower color) and juice vesicles (Fruit color), while a minus sign (−) indicates no visible coloration. A ± sign indicates that either the tree appeared chimeric and produced some colored and colorless fruit, or had visible coloration in immature fruit, but lost that color as it matured. The measured levels of anthocyanins, acidity (pH) and total sugar content (Brix) of the juice from mature fruit is shown. The chlorophyll content (SPAD value) and porosity readings (Stomatal conductance) from leaves of each event is also shown. Supplementary Table 1 includes graphs with the standard deviation values for analyzed samples.

Multiple independent events for each construct were grown to reproductive maturity in the greenhouse and had their growth and development monitored. The transgenic trees exhibited no obvious differences in either vegetative or reproductive growth patterns compared to wildtype Mexican lime trees. The immature trees carrying the CitVO1, CitUNK, PamMybA, and SlE8 constructs did not exhibit visible anthocyanin accumulation in root, stem or leaf tissues, but some of the *CitWaxp-Ruby* events had newly developed leaves that displayed visible purple coloration. This coloration, although clearly noticeable in new growth, disappeared as the leaves matured (Supplementary Figure 2). At approximately three or more years of age, the trees began to flower and fruit, which is typical for the Mexican lime cultivar.

**FIGURE 2:**
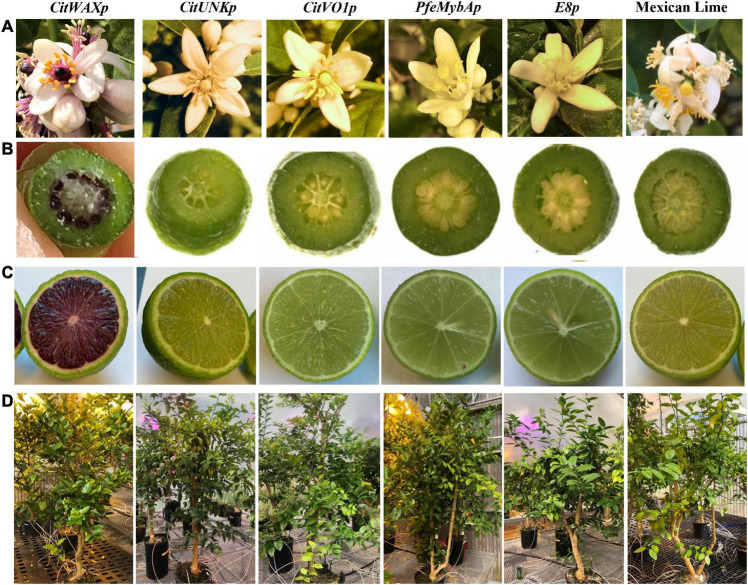
Visible phenotypes within the transgenic Mexican lime trees. **(A)** Coloration observed in wildtype and transgenic flowers. **(B)** Appearance of an immature fruit approximately 2 weeks after flower petal drop. **(C)** Mature fruit appearance approximately 3 months after flower petal drop. **(D)** Representative tree from each promoter-*Ruby* construct.

The *CitWaxp-Ruby* transgenic trees often produced flowers with pink or purple stigmas, styles, filaments and sometimes petals (Figure 2A), but none of the other transgenic trees carrying the other promoter constructs, nor did wildtype Mexican lime have this coloration. If cut open, the immature fruit the *CitWaxp-Ruby* transgenic events displayed a purple interior, while no coloration was visible in this tissue of wildtype or the other transgenic events (Figure 2B). The *CitWaxp-Ruby* transgenic events that exhibited a purple-colored stigma usually generated colored fruit (Figure 2C) and the strength of the pink or purple coloration of the flower and/or young leaves correlated well with the intensity of the coloration observed in the developing fruit. Although anthocyanins are known to transiently accumulate within the young shoots, leaves and floral tissues of some lemon cultivars [*Citrus limon* (L.) Burm. f.], citron (*Citrus medica* L.), and Rangpur lime (*C. limonia* Osbeck), this does not occur in Mexican lime (Rodrigo et al., 2013; Fabroni et al., 2016).

Within the *CitWaxp-Ruby* transgenic fruit, the pigmentation was only visible within the juice sac tissues, while coloration was not observed in the flavedo or albedo fruit peel tissues (Figure 2C). The seeds of these transgenic fruit also accumulated visible anthocyanins within the seed coat at the chalaza end (Supplementary Figure 3A). Seed germination was normal, and the transgene was inherited within the progeny as confirmed with genomic PCR (Supplementary Figure 3B). The transgenic events carrying the constructs with the *CitUNK*, *CitVO1*, *PamMybA*, or *SlE8* promoters fused to *Ruby* did not generate colored floral or fruit tissues, suggesting that these promoters failed to confer substantial expression in transgenic Mexican lime (Figure 2C, Table 1, and Supplementary Table 1).

Droplet digital PCR (ddPCR) was utilized to estimate the transgene copy number in the transgenic events (Table 1). The events carried between 1 and 9 copies of the *nptII* selection marker transgene with approximately 56% of the tested transgenic events carrying a single copy of the selection marker transgene. The overall growth and development of the transgenic trees did not appear to be impacted by the high transgene copy number. The growth and reproductive maturation for the high copy lines [e.g., CitVO1 1-1 (6.6 copies) and CitWax 9-37 (8.7 copies)] was indistinguishable from the single copy events (e.g., CitUNK 4-5, PamMybA 5-3A, and CitWax 7-2A) and wildtype Mexican lime trees (Figure 2D).

### Physiological assessment of the transgenic trees

All transgenic trees grew normally in the greenhouse and appeared similar to wildtype Mexican lime trees. The only noticeable difference in vegetative growth was visible anthocyanin accumulation within the young shoots of some of the *CitWaxp-Ruby* transgenic events. Given that anthocyanin accumulation in transgenic citrus has previously associated with suboptimal growth (Dutt et al., 2016), a chlorophyll meter (SPAD-502, Spectrum Technologies, Inc. Aurora, IL, United States) and leaf porometer (SC-1, Meter Group Inc.) were employed to investigate the physiological status of the transgenic trees in further detail. SPAD values, which are proportional to leaf chlorophyll content, are calculated from the transmission of red light at 650 nm (which is absorbed by chlorophyll), and the transmission of infrared light at 940 nm (which is not absorbed by chlorophyll). The porometer estimates stomatal conductance by measuring water loss from the leaf, which is dependent on the rate of transpiration. Although some variability was noted in these measurements, they were all generally within a range similar to that of the wildtype Mexican lime trees (Table 1 and Supplementary Table 1). The variation observed did not correlate with the transgenic construct type, transgene copy number, or anthocyanin content. The introduction of a *Ruby*-containing T-DNA into the Mexican lime genome therefore did not appear to affect nitrogen management or transpiration rates compared to the wild-type control.

### Characterization of the transgenic fruit

Brix is defined as the percent of sucrose and other total soluble solids by weight in a solution and is a measure of general sweetness of fruits and their juices. This includes compounds such as organic acids, soluble amino acids, and other miscellaneous compounds, such as fat, minerals, alcohol, flavonoids (Vitamin C and Vitamin A), as well as the total sugar content. The Brix value is used as a measure of maturity, flavor, and level of sweetness in fruits that helps determine the time of harvest, sales, and processing (Ball, 2006; Kleinhenz and Bumgarner; Lannes et al., 2007). A standard Brix assay was used to evaluate the sweetness of the juice produced from the transgenic trees. Mature ripe fruit were harvested just as they began turning yellow and the degrees Brix of the juice was measured (Table 1 and Supplementary Table 1). Wildtype Mexican lime fruit had juice with a 7.31 ± 0.20 degrees Brix and the juice from a blood orange was as expected sweeter with a 11.74 ± 0.20 degrees Brix. The transgenic events all produced juices with 6.6 to 8.8 degrees Brix, similar to wildtype Mexican lime and distinctly lower levels than blood orange (Table 1 and Supplementary Table 1). The degrees Brix of the *CitWaxp-Ruby* events ranged from 6.6 to 8.6, very similar to the range exhibited by Mexican lime and the other transgenic events (Table 1 and Supplementary Table 1). Taken together, the transgenic fruit all had similar Brix levels to that observed in Mexican lime, indicating that transgenesis and the expression of Ruby in the fruit did not significantly alter the overall sweetness of the limes.

Citrus flavor is influenced by the balance between sugars and acidity, mainly due to citric acid accumulation. As citrus fruit ripen, the acidity decreases while the sugars increase, so the amount of citric acid is among the major determinants of fruit maturity and the overall flavor (Strazzer et al., 2019). The pH of the transgenic lime fruit juices was measured to quantify their acidity. Table 1 shows that the pH of the transgenic events ranged from 1.9 to 2.4 which was very similar to that of wildtype Mexican lime with a pH of 2.1 but is significantly lower than the juice from the blood orange with a pH of 3.4 (Table 1). These results are in general agreement with lime juice typically having a pH between 2.00 to 2.35 as listed by Clemson University which published the PDF entitled “pH Values of Common Foods and Ingredients.” The *CitWaxp-Ruby* transgenic events had a pH similar to Mexican lime and the other transgenic events, indicating that the expression of Ruby within the fruit, and anthocyanin accumulation, did not noticeably affect the pH of the juice.

**FIGURE 3:**
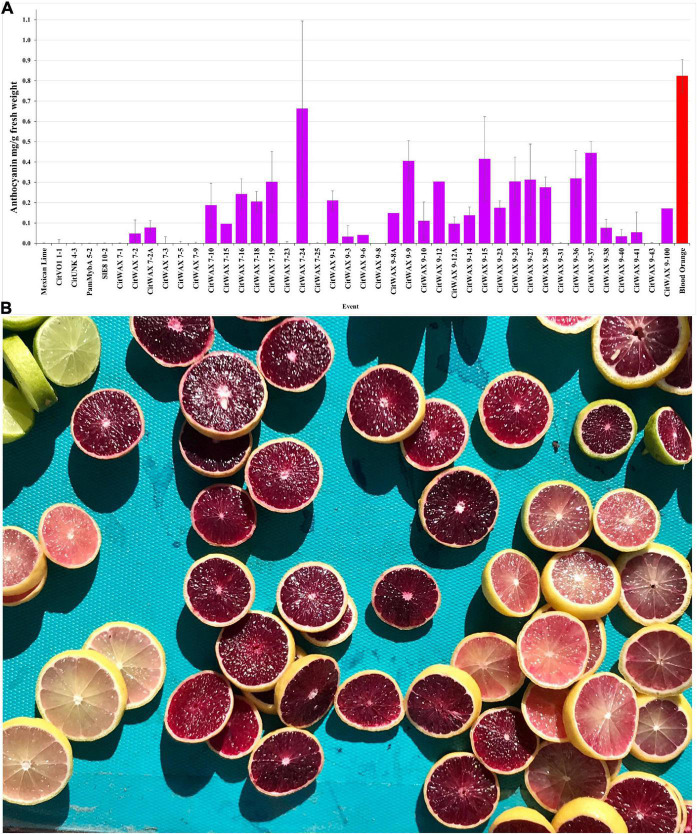
**(A)** Anthocyanin accumulation in Mexican lime fruit. Juice was collected from fruit and the anthocyanin content was determined spectrophotometrically. Wildtype Mexican lime (far left) and Moro blood orange (far right, red bar) are shown as comparators for the transgenic events (middle). A single representative event is shown for the CitVO1, CitUNK, PamMybA, SlE8 transgenic trees, while all of the characterized CitWax events are shown (fuchsia bars). **(B)** Mexican lime fruit derived from the *CitWax-Ruby* construct exhibited fruit of multiple purple hues. For comparison, wildtype Mexican lime fruit is shown in the upper left corner.

The anthocyanin content within the lime fruit juice from the transgenic trees was measured spectrophotometrically and compared to Moro blood orange and wildtype Mexican lime juices. The anthocyanin content was measured at wavelengths of 530 and 657 nm and the results are expressed as mg/g fresh weight as previously described (Neff and Chory, 1998; Chu et al., 2013). Wildtype Mexican lime has undetectable levels of anthocyanins in this assay, while Moro blood orange has 0.824 mg/g fresh weight. The *CitVO1p*-, *CitUNKp*-, *PamMybA1p*-, and the *SlE8p*-*Ruby* transgenic events did not have colored fruit (Figure 2C, Table 1, and Supplementary Table 1) and lacked detectable anthocyanin values in the assay, similar to wildtype Mexican lime. The *CitWaxp-Ruby* events displayed a variety of pink, fuchsia or purple hued fruits (Figures 2C, 3A,B, Table 1, and Supplementary Table 1) and a few events had undetectable anthocyanin coloration. The *CitWaxp-Ruby* events that had visible pigmentation of their fruit had anthocyanin values that ranged from 0.03 to 0.66 mg/g fresh weight, with the higher accumulating events having up to 75% of the level of anthocyanins found in blood oranges. The results also indicate that transgenic events with multiple copies of the introduced transgenes tended to exhibit more strongly pigmented fruit. Examples include CitWax event 7-24 (6.3 copies) with 0.633 mg/g fresh weight or 75% of the Moro blood orange value; event 9-37 (8.7 copies) and an absorbance of 0.455 mg/g fresh weight or 54%; and event 9-9 (6.6 copies) with absorbance of 0.406 mg/g fresh weight or 48% of the level found in blood orange. Conversely, those lines with one or two transgene copies, such as 7-5 and 9-40 provided a lightly colored fruit had anthocyanins levels that were below 5% of that found in blood orange. Seventeen of the top 20 anthocyanin accumulating events had two or more transgene copies.

The events that have low levels of anthocyanin in the juice, often had fruit that display a variable pattern of pigmentation (i.e., part of the fruit may be colored, while another part may not). In fact, for some events, there is a distinct lack of color surrounding the center of the fruit when sliced horizontally (Supplementary Figure 4), indicating a loss of anthocyanin in this area. Whether this is due to lack of expression in this region of the tissue or breakdown of the anthocyanins is unknown. *CitWaxp-Ruby* events 9-8 and 9-31 were unique in that they produced young fruit with easily visible anthocyanin pigmentation, but that pigmentation became weak or disappeared as the fruit matured (Supplementary Figure 5), and anthocyanin accumulation was not detected in the assay of their mature fruit (Figure 3A). The *CitWaxp-Ruby* events of potential commercial interest include those with low copy T-DNA inserts that exhibit substantial anthocyanin accumulation levels including CitWax 7-2A, 7-10, 9-1, and 9-10 (Supplementary Figure 6).

**FIGURE 4:**
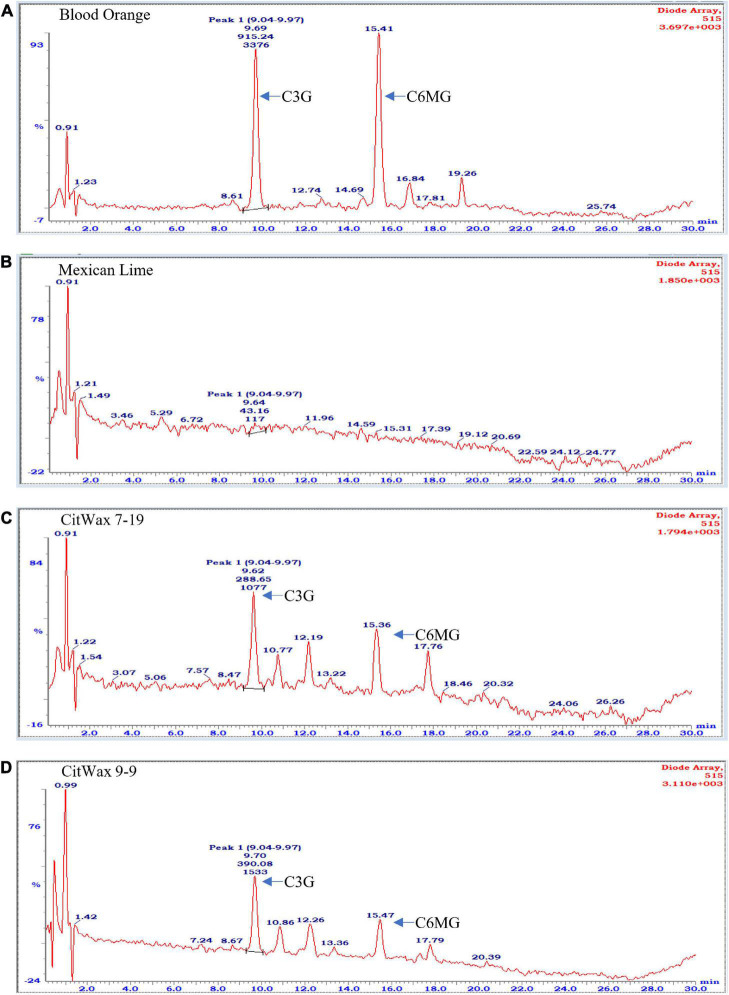
Chromatographic profile of anthocyanins found in citrus fruits. **(A)** Commercially bought Moro blood orange. **(B)** Wildtype Mexican lime. **(C)** Transgenic CitWax 7-19. **(D)** Transgenic CitWax 9-9. Cyanidin 3-glucoside (C3G) and cyanidin 3-(6′′-malonyl)- β-glucoside (C3-6MG) provide the two strongest and peaks at approximately 9.6 and 15.4 min with UV-Vis absorption at 515 nm.

Flavonoids, including anthocyanidins, flavanols, flavanones and isoflavones are abundant compounds found in citrus fruit (Gattuso et al., 2007). Cyanidin 3-glucoside (C3G) and cyanidin 3-(6′′-malonyl)- β-glucoside (C3-6MG) are among the anthocyanins commonly found in citrus (Lee, 2002) and are abundant pigments in blood oranges (Dugo et al., 2003; Scordino et al., 2015). The anthocyanin profile in blood orange detected by our HPLC analytical chromatographic analysis shows that C3G and C3-6MG are easily recognizable peaks within the UV-vis spectra at 515 nm. The juice from selected transgenic fruit was examined using these same conditions and the results were compared to blood orange and wildtype Mexican lime fruit samples. As shown in Figure 4A, the C3G and C3-6MG peaks are prominent at 9.69 and 15.41 min, respectively (Figure 4A), consistent with previously published results (Lee, 2002; Scordino et al., 2015). Wildtype Mexican lime fruit juice exhibited no discernable peaks at these elution timepoints (Figure 4B), with similar seen in representative samples from the *CitVO1p*-, *CitUNKp*-, *PamMybA1p*-, and the *SlE8p*-*Ruby* transgenic events (Supplementary Figure 7), which is consistent with their fruit not exhibiting visible anthocyanin pigmentation.

**FIGURE 5:**
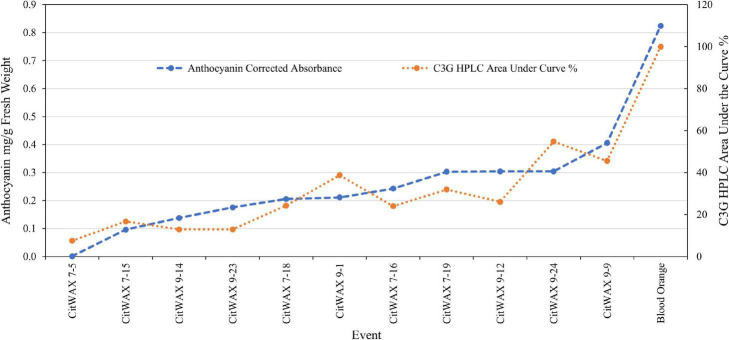
Comparison of anthocyanin absorbance content (Table 1) vs. chromatographic profile area under the curve values for cyanidin 3-glucoside (Figure 4) of select *CitWaxp-Ruby* fruits and blood orange control.

In contrast, samples from the *CitWaxp-Ruby* events exhibit a series of novel peaks, and the two strongest elute at 9.70 and 15.47 min for the CitWax 7-19 event and 9.62 and 15.36 min for the CitWax 9-9 event (Figures 4C,D). These two strongest peaks correspond to C3G and C3-6MG based on the blood orange trace. Interestingly, in the *CitWaxp-Ruby* events, the strongest peak is C3G followed by the C3-6MG, while in the blood orange sample, this is reversed. The CitWax transgenic events also exhibit three other prominent peaks between 10 and 18 min that are not present in wildtype Mexican lime fruit juice. We hypothesize that this shift in peak strength and the presence of other peaks not seen in the blood orange profile is likely due to the unique metabolism of the lime fruit as compared to that of the sweet orange. We performed a similar analysis for 11 other *CitWaxp-Ruby* events and observed similar results (Supplementary Figure 7). These results demonstrate that the *CitWaxp-Ruby* events accumulate cyanidin glucosides (C3G and C3-6MG), while wildtype Mexican lime does not accumulate detectable levels of these compounds. Area under of the curve analysis was used to estimate the amount of cyanidin 3-glucoside (C3G) in the *CitWaxp-Ruby* events compared to the Moro blood orange. The events ranged from 7.5% (CitWax 7-5) to 55% (CitWax 9-24) of the amount of C3G in blood orange; these values correlated well with the anthocyanin levels measured using the spectrophotometric absorbance assay shown in Table 1 and Figure 3A. Results from the spectrophotometric absorbance assay for anthocyanin absorbance assay values (Table 1) were plotted against the under of the curve HPLC analysis (Figure 4). The trend of relative anthocyanin levels remains consistent where the low copy line 7-5 is observed as a weak expressor in the colorimetric assay and remains low in the HPLC relative value determination, while 9-24 a multicopy high expression line remains high for the HLPC relative value (Figure 5).

**FIGURE 6:**
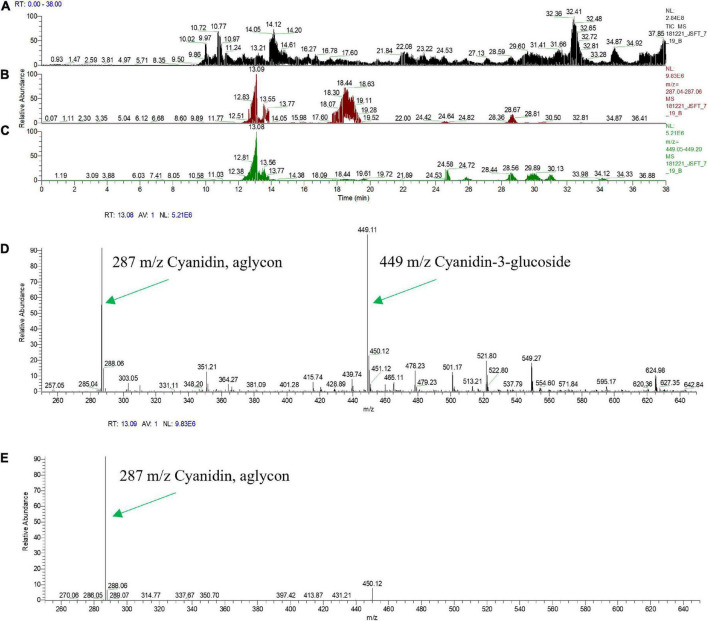
Mass Spectral Analysis (HPLC-MS-MS) profiles of fresh squeezed juice of line CitWax 7-19. **(A)** Top panel shows the ion capture of all charge in compounds from MS survey scan range of 200–2,000 m/z. **(B)** Second panel shows specific ion capture from 287.04 to 287.06 m/z. **(C)** Third panel shown specific ion capture from 449.05 to 449.20 m/z. **(D)** Forth panel shows the strongest specific peaks from the full MS1 survey scan range of 200–2,000 m/z at the retention time of 13.08 min. **(E)** Fifth panel shows MS2 of 449.11 m/z at retention time 13.09.

Multiple studies have previously examined blood orange juice anthocyanins using electrospray tandem mass spectrometry (MS-MS) and these efforts have provided the identification of the endogenous anthocyanin metabolites (Dugo et al., 2003; Scordino et al., 2015). Since anthocyanin aglycon compounds are typically unstable in the cellular environment, they are frequently observed in multiple glycosylated forms. MS-MS analyses enables the fragmentation of the anthocyanins and the identification of the specific glycosylated forms through the loss of the sugar moiety and the production of the positively charged aglycon. Representative CitWax events were analyzed along with Moro blood orange and wild-type Mexican lime samples using MS-MS in positive ion mode on a Thermo Orbitrap Elite instrument with a survey scan range of 200–2,000 m/z. The MS-MS spectra were reviewed for aglycone fragment ions and anthocyanin parent ions [e.g., m/z 287 ± 3 u Cyanidin aglycon m/z 449 ± 3 u Cyanidin-3-glucoside (C3G), at m/z 536 ± 3 u Cyanidin-3-malonyl glucoside (C3-6MG) and at m/z 596 ± 3 u Cyanidin-3- rutinoside (C3R)], (Figures 6A–C). Multiple anthocyanins were detected in this analysis including glycosylated forms of cyanidin, peonidin, delphinidin, and petunidin. The most abundant glycosylated forms detected in the *CitWaxp-Ruby* events include the glucoside, malonyl glucoside, and rutinoside forms of cyanidin (Table 2). Identification and peak assignment of anthocyanins were based on accurate mass measurements (within 10 ppm) of the calculated monoisotopic mass of both the MS1 anthocyanidin (Figure 6D) and its MS2 aglycone fragment (Figure 6E). The CitWax 7-19 sample is shown in Figure 6 and is representative of the *CitWaxp-Ruby* events (Supplementary Figure 8). The molecular weights of the three most abundant anthocyanidins and their sugar conjugates are shown in Table 2. Identification of selected metabolite peaks was performed by comparison of the retention time, the spectroscopic characteristics and observed mass spectra compared to that of blood orange juice (Supplementary Figure 8). The spectroscopic characteristics of the analytes detected in the blood orange juice samples are consistent with previously published results (Dugo et al., 2003; Scordino et al., 2015). Mass spectra results of the transgenic *CitWaxp-Ruby* events verify the presence of multiple anthocyanin metabolites in the isolated juice of the colored fruit. The cyanidin and peonidin glucosides and malonyl glucosides are seen at detectable levels in the *CitWaxp-Ruby* events, where none was observed in wildtype Mexican lime. Also, the peonidin and delphinidin rutinosides are present in the *CitWax* events, but not observed in the Moro blood orange sample (Table 2).

**TABLE 2:** MS-MS identified anthocyanins.

Anthocyanins and glycoforms	Calculated m/z	Blood orange	Mexican lime	CitWax 9-9	CitWax 7-19
Cyanidin aglycon	287.056				
Glucoside (C3G)	449.108	4.4^5^	ND	9.6^5^	5.2^6^
Malonyl glucoside (C3-6MG)	535.109	4.5^5^	ND	5.1^5^	6.6^6^
Rutinoside (C3R)	595.166	ND	1.0^6^	1.2^6^	2.6^6^
Peonidin aglycon	301.071				
Glucoside	463.12	2.4^4^	ND	6.8^5^	3.6^6^
Malonyl glucoside	549.124	4.4^4^	ND	6.6^5^	3.9^6^
Rutinoside	609.182	ND	1.9^7^	5.2^6^	2.1^7^
Delphinidin aglycon	303.051				
Glucoside	465.103	4.6^4^	ND	ND	4.0^5^
Malonyl glucoside	551.104	1.0^5^	ND	ND	7.0^5^
Rutinoside	611.161	ND	2.4^6^	5.1^6^	6.5^6^
Petunidin	317.070				
Glucoside	479.123	ND	ND	ND	ND
Malonyl glucoside	565.123	ND	ND	ND	ND
Malonyl rutinoside	625.181	ND	2.0^6^	1.5^6^	7.8^6^

Values for each sample are absolute values of peak height from reconstructed ion current profiles for the m/z values of the glycosylated forms are indicated. ND, not detected. Superscript numbers indicate scientific notation.

To further characterize the *CitWaxp-Ruby* transgenic fruit, the nutrient composition was analyzed for events 7-18 and 9-9 and compared to wildtype Mexican lime juice. The results shown in Table 3 indicate that other than a reduction in fructose and glucose content, the nutritional content of the transgenic fruit was overall very similar to wildtype fruit. The observed reduced levels of fructose and glucose may be at least partially due to differences in the ripeness of the samples and can be partially offset by the observed modest increases in sucrose. This result is generally consistent with Brix data for these events being similar to wildtype (Table 1 and Supplementary Table 1). The differences in the sugar levels may also be due to environmental differences between the greenhouse grown transgenic samples compared to the field grown wildtype samples used in the analysis, which is supported by previously published results showing that environmental conditions can affect sugar production within the citrus fruit (Zhang and Ritenhou, 2016; Sadka et al., 2019).

**TABLE 3:** Nutrient compositional analysis of CitWax lines 7-18 and 9-9 compared to store bought wildtype Mexican lime juice.

Analyte	Wildtype	CitWax 7-18	CitWax 9-9	Units
Carbohydrate	7.80	8.20	7.38	g/100 g
Fat	0.88	0.90	0.91	g/100 g
Moisture	90.8	90.4	91.2	g/100 g
Protein	0.33	0.31	0.33	g/100 g
Fructose	0.32	0.28	<0.01	g/100 g
Glucose	0.22	0.09	<0.01	g/100 g
Sucrose	0.00	0.01	0.05	g/100 g
Vitamin A	N.D.	N.D.	N.D.	mcg/100 g
Vitamin C	32.5	19.4	20.8	mg/100 g
Calcium (CA)	0.59	0.29	0.33	mg/100 g
Copper (Cu)	0.02	0.01	0.01	mg/100 g
Iron (Fe)	0.02	0.07	0.05	mg/100 g
Magnesium (Mg)	5.50	5.57	5.40	mg/100 g
Phosphorus (P)	1.78	2.19	2.54	mg/100 g
Potassium (K)	16.7	19.3	17.4	mg/100 g
Selenium (Se)	<0.5	<0.5	<0.5	mg/100 g
Sodium (Na)	0.55	0.36	0.37	mg/100 g
Zinc (Zn)	0.10	0.11	0.12	mg/100 g

Analysis was conducted by the USDA-AMS National Science Laboratory. <0.01 and <0.05 values mean below the limit of accurate detection. Mexican lime control fruit was field grown, store bought. CitWax lines 7-18 and 9-9 were greenhouse grown.

## Materials and methods

### Plant material and promoter isolation

Binary plasmid construction was as previously described (Dasgupta et al., 2020). The *GUS* gene was replaced by the *Ruby* (*CsMybA1*) coding sequence. The *Ruby* (*CsMybA*/*MoroMybA*) genomic sequence including native introns was synthesized (GenScript USA Inc.) to remove native restriction sites and to aid in cloning (Dasgupta et al., 2017). The complete *Ruby* sequence is available in Supplementary Text 1 of a previous publication, (Dasgupta et al., 2017). The nucleotide sequences for the tested promoters are available from the GenBank under the following accession numbers *CitWaxp* (GenBank MK012380), *CitUNKp* (GenBank MK012381), *CitVO1p* (GenBank MK012383), (Belknap et al., 2015), *SlE8p* (GenBank KJ561284), and *PamMybAp* (GenBank MK012385). Each construct is derived from the pCTAGII-GUSPlus binary vector (GenBank MG818373) contains a unique promoter of interest that is used to express *Ruby*, along with the *codA-nptII* selection marker for transgenic tissue selection (Figure 1A).

### Production of transgenic Mexican lime

Mexican lime (*C. aurantifolia*) juvenile tissue was used for hypocotyl transformation as previously described (De Oliveira et al., 2009). Certified Mexican lime seed was obtained from Lyn Seed^1^. The seed was removed of its outer seed coat and surface sterilize the by soaking in 20% bleach for 20 min. Seeds were then rinsed three times in sterile water and 3–5 seeds were placed on citrus seed germination medium (4.4 g/L MS salts with vitamins, 25 g/L sucrose, adjusted to a pH of 5.8 with 1 N NaOH, 8.0 g/L Bacto Agar) and incubated in the dark at 28°C for 4–5 weeks. Etiolated seedlings that were 12–15 cm long were used for transformation. A 25 mL *Agrobacterium* culture was prepared in YEP medium with 50 mg/l kanamycin and 200 μM acetosyringone and incubated at 28°C shaker at 225 rpm overnight. The *Agrobacterium* culture was then centrifuged at 3,600 × *g* for 10 min at 22°C, the supernatant poured off and the pellet resuspended in liquid inoculation medium (4.4 g/L MS salts with vitamins, 30 g/L sucrose, adjusted to a pH of 5.8 with 1 N NaOH. After autoclaving add, 200 μM acetosyringone, 400 μl myo-inositol 100 mg/L, 1 mg/ml thiamine-HCL, 1 mg/mL pyridoxine, 1 mg/mL nicotinic acid and 1 mg/mL BAP) and adjusted to an OD_600_ of 0.2–0.4.

Young, healthy stems were cut into pieces approximately 10 mm in length with the ends cut with at least 45° angle so that each angle will be in the opposite direction of the other. The segments were incubated in the *Agrobacterium* suspension for 10 min and then blotted and allowed to dry on sterile filter paper for 5 min. They were then placed on co-cultivation medium [1.21 g/L Broadleaf Tree Basal Medium (B1396 – Phytotechnology Labs), 30 g/L sucrose, adjusted the pH 5.8 with 1 N NaOH, and then 8.0 g/L Bacto Agar. After autoclaving, 200 μM acetosyringone, 400 μl/L, myo-inositol 100 mg/L thiamine HCL, 1 mg/mL pyridoxine, 1 mg/ml, nicotinic acid, 1 mg/mL glycine 1 mg/L BAP, 1 mg/ml 2.4-D, and 1 mg/mL, NAA – Sigma-Aldrich] in sterile petri dishes and kept in the growth chamber at 28°C for 2 days in the dark. The infected explants were then transferred to selection regeneration medium (SRM; same as the co-cultivation medium with 70 mg/L of kanamycin added). They were incubated for 2 weeks in dark and then transferred to an incubator at 28°C/16 h light and 24°C/8 h dark. The explants were transferred to fresh SRM every 2–3 weeks.

Primary Mexican lime transformants regenerated on SRM with 50 mg/L kanamycin. Individual green shoots from explants were excised when approximately 10–12 mm in length and grafted onto Carrizo seeding rootstocks. Once the grafts were established in tissue culture, the plantlets were transferred to soil in a growth chamber at 16/8 light dark cycle and 28°C under a humidity dome and allowed to grow for another 4 weeks before being transferred to the greenhouse where they were maintained with a mist spray for 2 weeks and then allowed to harden to greenhouse conditions. Greenhouse conditions were 16/8 light dark cycle and 26°C.

### Polymerase chain reaction

Genomic DNA was extracted by grinding a 1 cm^2^ piece of citrus leaf in 400 μL of buffer (200 mm Tris–HCl pH 7.8, 250 mm NaCl, 25 mm EDTA, and 0.5% SDS). After centrifugation and isopropanol precipitation, the pellet was washed with 70% ethanol and resuspended in 50 μL of water with 1 mM RNase A. PCR amplification was performed using 0.5 μL (∼50 ng) of genomic DNA in reactions with a total volume of 20 μL. Platinum Superfi (Invitrogen) reagents and conditions were used as directed by manufacturer. Sequences of the PCR primers that were used for amplification are shown in Table 4. Droplet digital PCR (ddPCR) was performed following the methods described in Collier et al. (2017). Sequences of the ddPCR primers and probes used are shown in Table 4.

**TABLE 4:** Primers and probes used in PCR and ddPCR.

Primer	Sequence	Amplicon
CitUNK4p 375 F60	GGACTCAGCAACCCTACCCAAGTG	1,058 bp
CitVO1p 400 F60	CACATGCACTAACTTAACCATATAGAGCTGTTGACC	1,080 bp
CitWaxp 490 F61	GGACGATTGTGTTACAGAGAGCATTTAATAAAGCACC	1,179 bp
SlE8p 800 F60	GGTTTAGTCCACAAGTTTTAGTGAGAAGTTTTGC	1,574 bp
PamMybAp 700 F60	CAGCGGAGTCTAACATCCTACGAATAAACCG	1,395 bp
Ruby 670 R60	GGGTAGTTTATGTGTATGCTATATGTTGCTCAACC	
codA ORF 70 F60	CATCTGCAGGACGGAAAAAT	1,137 bp
codA ORF1137 R60	GATAATCAGGTTGGCGCTGT	
CsDehydrin F2	GCCACCGAGTTTGAGAAAG	134 bp
CsDehydrin R2	GAGCTAGAGCTGCTGGTG	
Cs1.1g026736m.g (FAM)	ATGTCTCTGAGCCTCAGCCA	
1116_nptII-F3	ACGTTGTCACTGAAGCG	100 bp
1117_nptII-R3	ATGGATACTTTCTCGGCAG	
nptII_Probe3 (HEX)	TCTCCTGTCATCTCACCTTGCTC	

### Imaging of plant tissues

Photographs of the plants and their tissues were recorded using a Nikon D7000 digital camera with an AF Micro Nikkor 60 mm 1:2.8 D lens or AF-S Nikkor 18–70 mm DX lens (Nikon Inc., Melville, NY, United States) under tungsten lamps (Philips, 120 V, 300 W). The camera was set manually for all parameters including ISO sensitivity, focus, f-stop and time. A photography gray card was used as a reference to get the correct exposure. The callus images in petri plates were observed and photographed in a Leica MZ16-F (Leica Microsystems, Inc., Buffalo Grove, IL, United States) stereo zoom light microscope equipped with a QImaging Retiga 2000 R fast cooled, digital color camera.

### Juice extraction

Ripe lime fruit (just as they were beginning to turn yellow) were harvested from the trees as they became available. The fruit was rolled on a flat surface and then cut horizontally in half. The juice was squeezed into a beaker using a handheld 8-inch aluminum lime juicer. The juice was then strained through a fine mesh metal strainer and poured into 1.5 mL plastic tubes. These tubes are then spun in a centrifuge at 13,500 RPM for 5 min. The juice was the decanted into a clean beaker and syringe filtered (0.2 μm nylon filter) into 1.5 ml tubes. Tubes were labeled and stored in a −35°F freezer in the dark. The Moro blood orange control juice was obtained from field grown, store bought source that had relatively uniform pigmentation. A set of 10 oranges were bought together, juiced together, filtered, aliquoted, and frozen −35°C. This homogenized juice was used as the blood orange control for all subsequent experiments.

### Spectrophotometric (colorimetric) anthocyanin assay

Total anthocyanin levels in the juice were determined using methanolic HCl and measured spectrophotometrically at wavelengths of 520 and 700 nm as described by Chu et al. (2013). All samples were measured in duplicates with six biological replicates for each independent line when available. The anthocyanin content is expressed as absorbance (Abs) in mg/g fresh weight as previously described (Neff and Chory, 1998; Chu et al., 2013). Two 500 μL samples of 0.2 μm filtered juice was prepared per citrus event sample examined. The first sample had HCl added to achieve a pH = 1.0. The second sample had NaOH added to achieve a pH of 4.5. The sample was vortexed to mix it and then let stand for 10–15 min covered or in the dark. A volume of 250 μL sample was loaded per well into a 96 well plate and a SpectraMax Plus 384 spectrophotometer (Molecular Devices Corporation, CA, United States) was used to read the absorbance. Samples were assayed in triplicate (i.e., 3 × pH 1.0 and 3 × pH 4.5). A 50% methanol 50% water solution was used as a blank. Sample absorbance from 400 to 710 nm in 10 nm steps was recorded and analyzed using SoftMax Pro software (Molecular Devices, CA, United States). The anthocyanins are calculated as cyanidin-3-glucoside equivalents, mg/L, using the equation:

where A = (A 520 nm − A 700 nm) pH 1.0 – (A 520 nm − A 700 nm) pH 4.5.

A × MW × DF × 103/ε × l

MW (molecular weight) = 449.2 g/mol for cyanidin-3-glucoside (C3G);

DF = dilution factor (1:10);

l = path length in cm;

ε = 26,900 molar extinction coefficient, in L × mol^–1^ × cm^–1^, for C3G;

1,000 = factor for conversion from g to mg.

### Chromographic analysis of anthocyanins using HPLC at 515 nm

Analysis of anthocyanins was performed using an Alliance Waters 2695 Separation Module (Milford, MA, United States) coupled to a 996 PDA detector. The system is equipped with a Luna 5u C18 (Number 28; 150 mm × 3.0 mm) column with a guard column of the same material. Temperature of the column and pre-column is maintained at 40°C with an isocratic mobile phase consisting of the monobasic solution. The flow is 0.100 mL/min and injection volume of standards, control and samples. The total run time is 20 min for a blank, and 30 min for the samples. The analysis was conducted at 40°C with a 3.0 mm × 150 mm, 5 μM, Luna C-18 (2) column (Phenomenex, Torrance, CA, United States) employing a binary gradient of 2% formic acid: acetone (time 0–15 min, 2–12% acetone; time 15–20, 12–22% acetone; time 20–25, 22% acetone; time 25–30, 2% acetone). The flow rate was 1 mL/min. Masslynx (version 4.1) is used to control the HPLC system and for data analysis. Standard curve and control are injected at the beginning and at the end of the set of samples. Integration of individual peak areas is done using Quanlynx, and quantification is based on external calibration curves of anthocyanins at the wavelength of 515 nm.

### Anthocyanin analysis using HPLC-mass spec-mass spec

Reverse phase HPLC was accomplished with an Eksigent Ekspert nanoLC 425 fitted with a PicoSlide column switching device and three Reprosil-PUR C18-AQ, 3 μM 120A; 105 mm PicoChip columns (New Objective, Woburn, MA, United States). Samples were loaded directly onto the column with 400 nL/min 2% for 60 min. Column elution was at 400 nL/min with a program of 3% for 6 min, followed by a 36-min linear ramp to 25%, followed by a 16 min linear ramp to 50%, followed by a 2 min linear ramp to 3%. Sample elution was followed by a sawtooth column wash step with four cycles to 90%, linear ramp from 5 to 90% over 3 min, hold at 90% for 3 min, then ramp to 5% over 3 min, followed by 24 min at 2%.

Mass Spectral analyses were performed in positive ion mode on a Thermo Orbitrap Elite (Thermo Fisher, Waltham, MA, United States), with mass resolution set to 30,000 for survey scans and, and fragment ion scans. Samples were loaded with an Eksigent Ekspert nanoLC 425 (SCIEX) directly onto a PicoChip C18 nanoflow column mounted on a PicoSlide ion source (New Objective, Woburn, MA, United States). The HPLC was programed with 400 nL/min 2% for 60 min and eluted accordingly. Mass Spectral Analysis was performed in positive ion mode on a Thermo Orbitrap Elite with a survey scan range of 200–2,000 m/z with MS2 spectra being collected for the most intense ions in a given survey scan. Electrospray source voltage was set to 3.5 kV and capillary inlet temperature set to 275°C. Fragmentation was performed in CID mode with 30 V normalized collision-induced dissociation (CID) of the top three most intense ions from the survey scan. Data were processed with Thermo Xcalibur software. The presence of aglycones was observed using reconstructed ion current profiles, and glycosylation was determined by m/z measurement of intact compounds, confirmed by MS-MS fragmentation of those compounds. Compounds detected were primarily mono- and di-saccharides of the respective anthocyanins. Anthocyanins detected were cyanidin, peonidin, delphinidin, petunidin, and another anthocyanin differing in molecular weight from delphinidin by 0.04 Da. The mass of 303.09 m/z is consistent with the mass of 5-Methyl-6-hydroxyluteolinidin 6,7,3′,4′-tetrahydroxy-5-methoxy-flavylium, however, this will require further investigation to confirm. The data was processed with Thermo Xcalibur software.

### Plant physiological measurements

Leaves were measured for chlorophyll content using a SPAD-502Plus chlorophyll meter (Spectrum Technologies, Plainfield, IL, United States). SPAD values are calculated by the meter from the transmission of red light at 650 nm (which is absorbed by chlorophyll), and the transmission of infrared light at 940 nm (which is not absorbed by chlorophyll). All measurements were made at a central point on the leaf between the midrib and leaf margin for three randomly selected leaves per tree and averaged to generate a single value. A porometer (SC-1, Meter Group Inc.) was used to assess stomatal conductance in leaves. All measurements were made at a central point on the leaf between the midrib and leaf margin for three randomly selected leaves per tree and averaged to generate a single value. All trees including the control Moro blood orange and Mexican lime were greenhouse grown for analysis.

### Juice analysis

The nutrient analysis of juice samples (175 mL) was conducted by the USDA-AMS National Science Laboratory (Gastonia, NC) for a comprehensive 17 component analysis. Samples were analyzed for Carbohydrate, Fat, Moisture, Protein, Fructose, Glucose, Sucrose, Vitamin C, Calcium (CA), Copper (Cu), Iron (Fe), Magnesium (Mg), Phosphorus (P), Potassium (K), Selenium (Se), Sodium (Na) and Zinc (Zn). Report ID #AU27038, AU27039, and AU27040. The National Science laboratory utilizes AOAC (Association of Official Analytical Chemists) methodologies for testing or a modified approach of an AOAC method. The laboratory is ISO/IEC 17025:2017 accredited and utilize methods of testing that are fit for purpose that have been validated http://www.eoma.aoac.org/#:~:text=AOAC%20is%20a%20leader%20in,and%20scientific%20information%20and%20opportunities.

The pH of the juices was determined using a Thermo Scientific Orion 3 Star pH Benchtop double junction micro pH meter. Due to the limited amount of juice available, pH readings were performed in 1.5 mL plastic tubes. Readings were taken in triplicate and the pH meter probe was thoroughly rinsed with purified water between readings.

A J47 refractometer (Rudolph Research Analytical, Hackettstown, NJ, United States) was used for juice Brix analysis. A 200 μL aliquot of purified water was used as a blank. A 200 μL sample of lime juice was used for analysis. Values were measured in triplicate and recorded. The refractometer prism was rinsed with water and then 50% ethanol between samples. The Mexican lime control fruit was field grown, store bought, while the CitWax lines 7-18 and 9-9 were greenhouse grown.

## Conclusion

The research presented demonstrates that the Moro blood orange *Ruby* transcription factor, when expressed using the *CitWaxp* fruit-preferential promoter, confers substantial anthocyanin accumulation within Mexican lime fruit. Somewhat surprisingly, the other tested fruit-preferential novel promoters, *CitVO1p, CitUNKp*, *PamMybAp*, and control *SlE8p*, did not express sufficient levels of Ruby to confer anthocyanin accumulation in fruit or other Mexican lime tissues (Figure 2). The molecular and physiological characterization of the transgenic trees showed that they all grew similarly to wildtype Mexican lime trees in the greenhouse. The *CitWaxp-Ruby* transgenic Mexican lime trees, in addition to accumulating anthocyanins within the fruit juice sac tissues, also frequently produced colored flower tissues (primarily the pistil and filaments) and occasionally within young emerging shoots and leaves. The anthocyanin accumulation in these events ranged from undetectable to as high as 75% of levels detected in the Moro blood orange via colorimetric spectrophotometry (Figure 3 and Table 1). It was noticed that the anthocyanin accumulation levels in the *CitWaxp-Ruby* transgenic events correlated to the transgene copy number of the event [higher copy events tended to have higher anthocyanin levels (Figure 3 and Table 1)].

To further investigate the transgenic fruit, the level of anthocyanins within lime fruit juice was examined using HPLC and MS-MS analyses (Figures 4, 5). The results establish that the *CitWaxp-Ruby* transgenic Mexican lime fruit contain cyanidin/peonidin glucosides and malonyl glucosides where not previously observed in wildtype Mexican lime. The observed levels of peonidin/delphinidin rutinosides in these events were also at higher levels than those found in the Moro blood orange. Nutritional analysis of the juice showed that 17 major components were within normal range and similar to the levels observed in wildtype Mexican limes (Table 4). A slight reduction in the sugar content was detected in this analysis, but the difference is likely to the greenhouse growing conditions in which the transgenic trees were grown. The fruit from the various transgenic lines were also examined for pH and Brix illustrating that they also were within the normal range of values for Mexican lime fruit (Table 1). Although the transgenic trees characterized in this study exhibited normal growth and fruit production, in our previous study, we had observed detrimental effects on two of our regenerated transgenic events that were darkly pigmented within vegetative tissues (Dasgupta et al., 2017). These plants displayed curled leaves, a lack of vigor, and stunted growth, in the greenhouse that appeared to correlate with apparent vegetative expression of a *mybA1* and substantial anthocyanin accumulation in leaves and other vegetative tissues (Dasgupta et al., 2017). In agreement with these observations, previous reports have shown that constitutive production of high levels of anthocyanins are toxic to the health of the transgenic plants and inhibit plant growth (Bradley et al., 1998; Stover et al., 2013; Dutt et al., 2016). Although the mechanism behind the reduction in plant growth is not fully understood, it is hypothesized that anthocyanin production may interrupt auxin transport, negatively impacting growth (Belknap et al., 2015). The research presented here demonstrated that utilizing a fruit preferential promoter to control *Ruby* expression, limits anthocyanin production to specific tissue types and generates trees that grew, matured and produced fruit similar to the wild-type control.

Previous studies have shown that blood orange cultivars require strong day-night temperature differentials for intense color formation in the fruit and therefore are dependent on the prevailing climate during fruit ripening for activation of the anthocyanin production (Dugo et al., 2003). However, the *CitWaxp-Ruby* transgene is not under environmental constraints and its tissue specific expression confers colored lime fruit by accumulating multiple different anthocyanin glycosides within the juice sac tissues. As anthocyanins exhibit antioxidant activity against harmful free radicals (Li et al., 2007; Reddy et al., 2007), act as bacteriostatic agents (Naz et al., 2007) and have been associated with the prevention of obesity and diabetes (Tsuda et al., 2003), this novel citrus fruit could be a beneficial addition to the human diet.

The *CitWaxp-Ruby* transgenic trees also exhibited novel coloration in the flowers and young leaves via anthocyanin overproduction. These lines could potentially be used as novel ornamentals, or the tissues could be used as a source of extracted anthocyanins to enhance the color and nutritional value of other foods. In addition, this research opens up the possibility for the development of other novel citrus cultivars that produce anthocyanin rich fruit under variety of environmental conditions. The *CitWax* promoter will be a potentially useful tool for engineering novel fruit traits within citrus and potentially other crops.

## Significance statement

Multiple tissue-specific promoters were tested for their ability to confer anthocyanin accumulation with *C. aurantifolia* fruit. The results presented demonstrate the ability to precisely genetic engineer anthocyanin accumulation in Mexican lime fruit.

## Data availability statement

The original contributions presented in this study are included in the article/Supplementary material, further inquiries can be directed to the corresponding author.

## Author contributions

JT conceived the project. JT, RT, and KD designed the experiments, analyzed the data, and wrote the manuscript. JT, RT, KD, JH, DH, LH, and RC performed the experiments. All authors approved the final manuscript.

## Footnotes

1
https://lyncitrusseed.com
