# Supplementary material for: Tissue-specific expression of Ruby in Mexican lime (C. aurantifolia) confers anthocyanin accumulation in fruit
Source: Front Plant Sci. 2022 Aug 8;13:945738. doi: 10.3389/fpls.2022.945738 (PMC9393592; doi:10.3389/fpls.2022.945738)

# Supplemental Figure S1

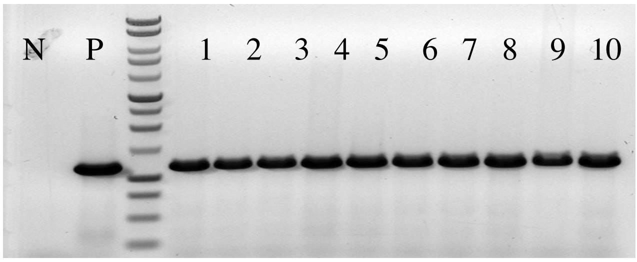


*CitWaxp-Ruby* (7 series)

1149 BP

CitWaxp 490 F61
GGACGATTGTGTTACAGAGAGCATTTAATAAAGCACC
CsMyba 670 R60
GGGTAGTTTATGTGTATGCTATATGTTGCTCAACC

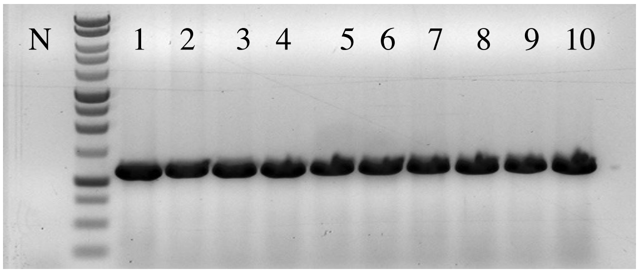


*CitWaxp-Ruby* (9 series)

1149 BP

CitWaxp 490 F61
GGACGATTGTGTTACAGAGAGCATTTAATAAAGCACC
CsMyba 670 R60
GGGTAGTTTATGTGTATGCTATATGTTGCTCAACC

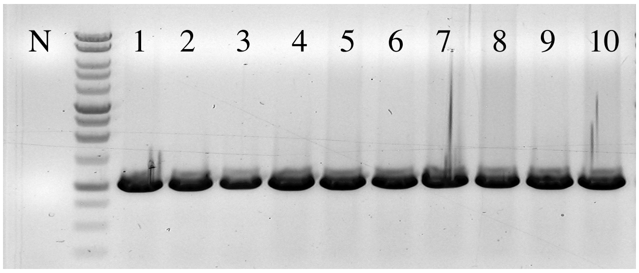


*CitUNKp-Ruby*

1058 bp

UNK4p 375 F60
ggactcagcaaccctacccaagtg
CsMyba 670 R60
GGGTAGTTTATGTGTATGCTATATGTTGCTCAACC

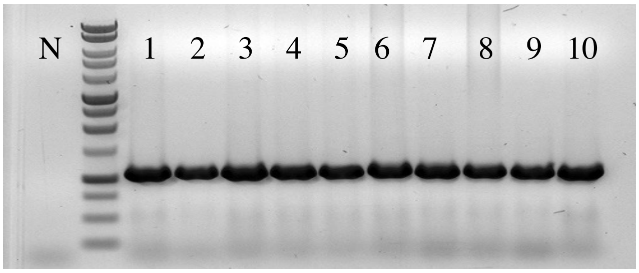


*CitVO1p-Ruby*

1080 bp

CitVO1p 400 F60
CACATGCACTAACTTAACCATATAGAGCTGTTGACC
CsMyba 670 R60
GGGTAGTTTATGTGTATGCTATATGTTGCTCAACC

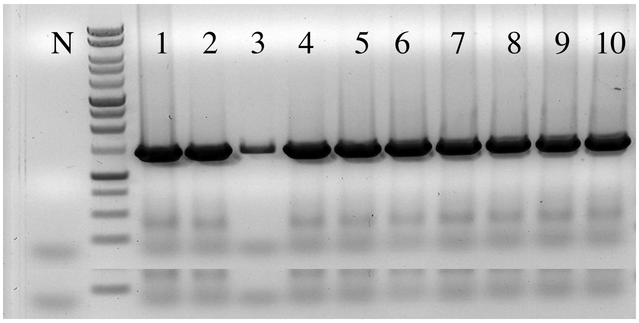


*PamMybAp-Ruby*

1395 bp

PfeMybAp 700 F60
CAGCGGAGTCTAACATCCTACGAATAAACCG
CsMyba 670 R60
GGGTAGTTTATGTGTATGCTATATGTTGCTCAACC

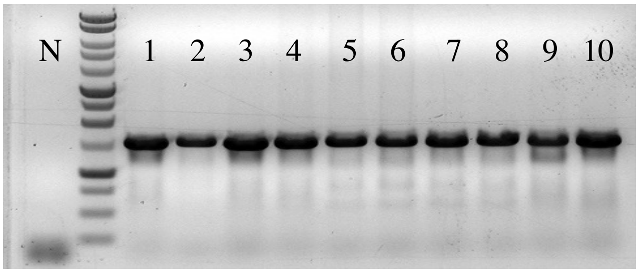


*SlE8p-Ruby*

1574 bp

E8p 800 F60
GGTTTAGTCCACAAGTTTTAGTGAGAAGTTTTGC
CsMyba 670 R60
GGGTAGTTTATGTGTATGCTATATGTTGCTCAACC

Supplemental Figure S2

Multi copy lines. High expression. Juvenile leaves expressing anthocyanin

9-23

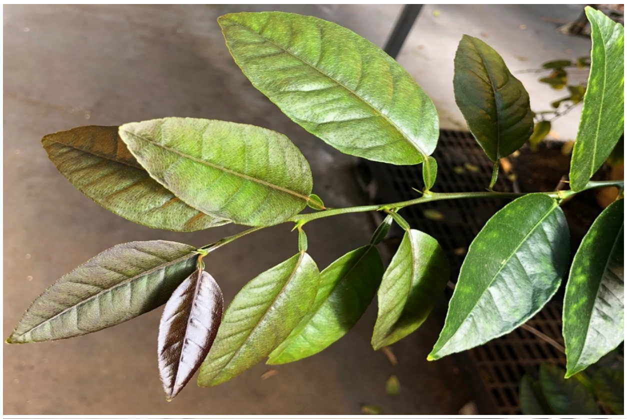

9-9

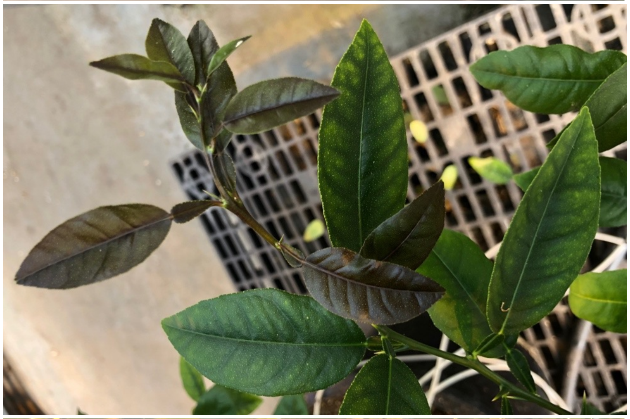

7-18

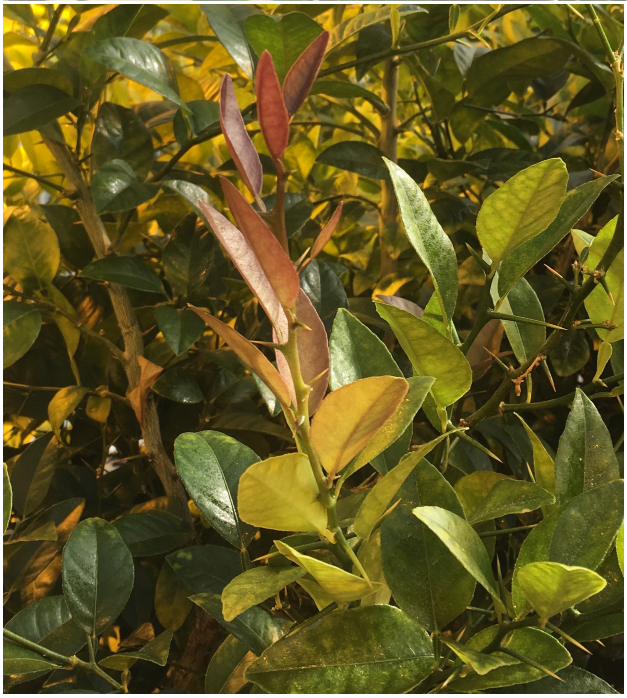

Supplemental Figure S3

A). Seed harvested from line 9-1. Purple color seen within the seed coat at the chalaza end .

B). Successful seed germination and heritable transmission of the *Ruby* transgene in CitWAX line 9-1

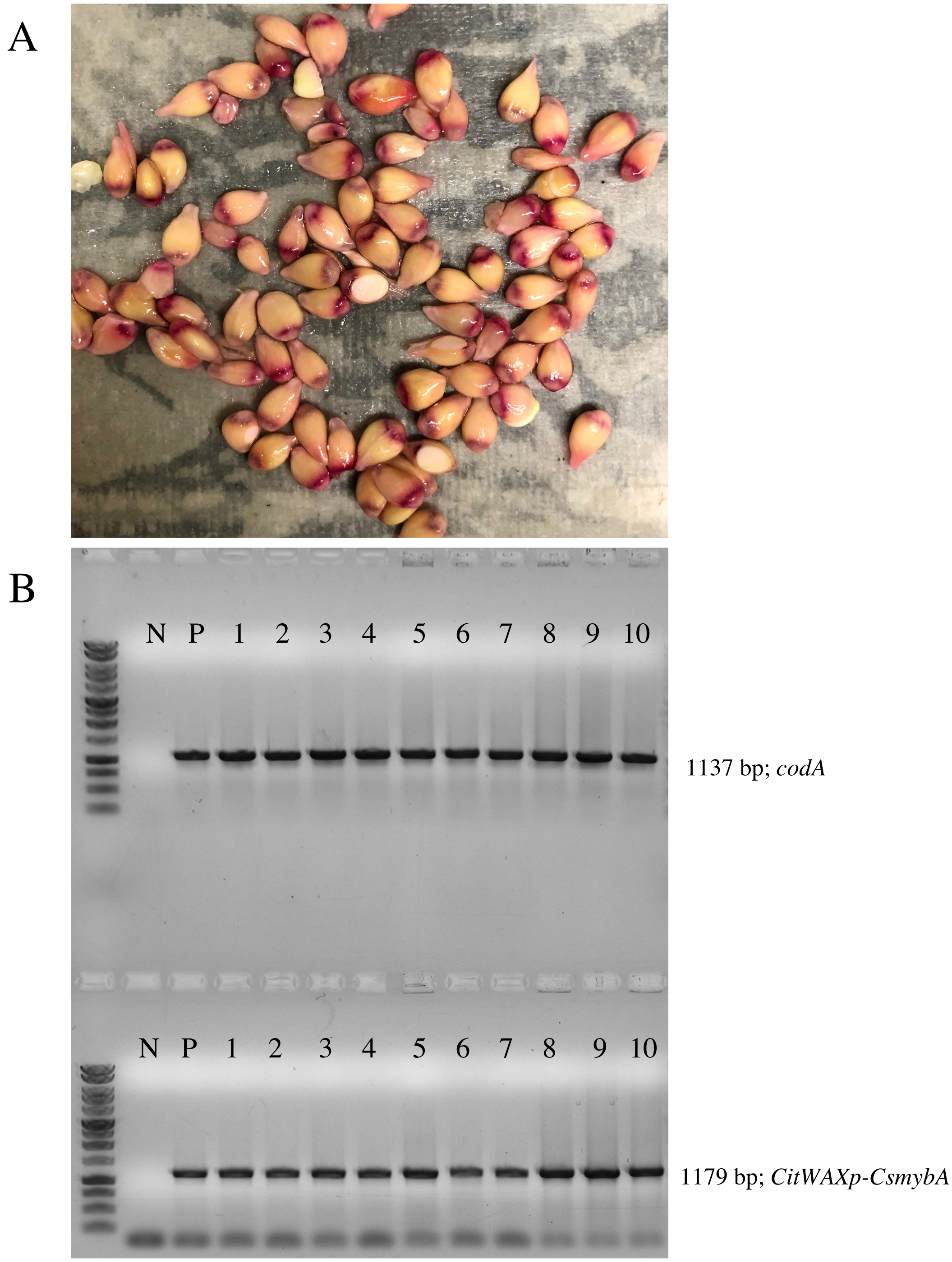

Supplemental Figure S4

Low copy limes.  Low expression.
Central clearing of color

9-40

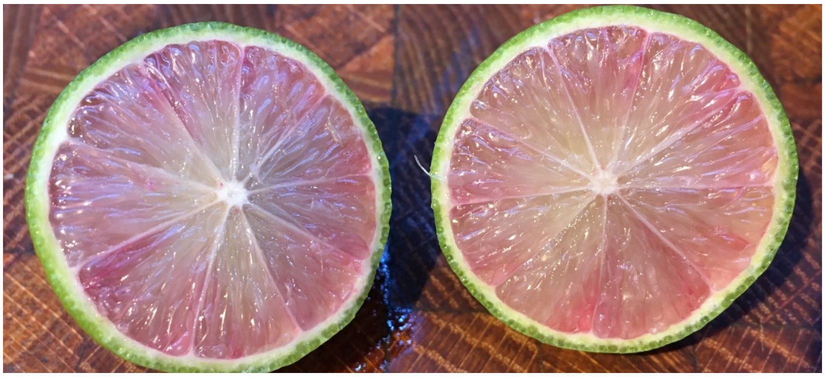

9-41

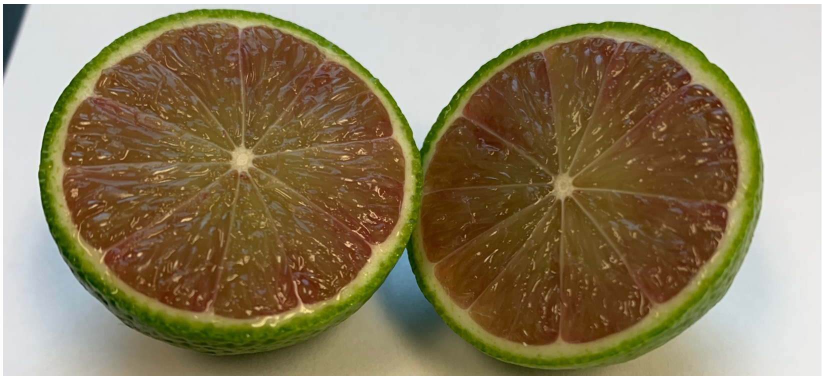

7-5

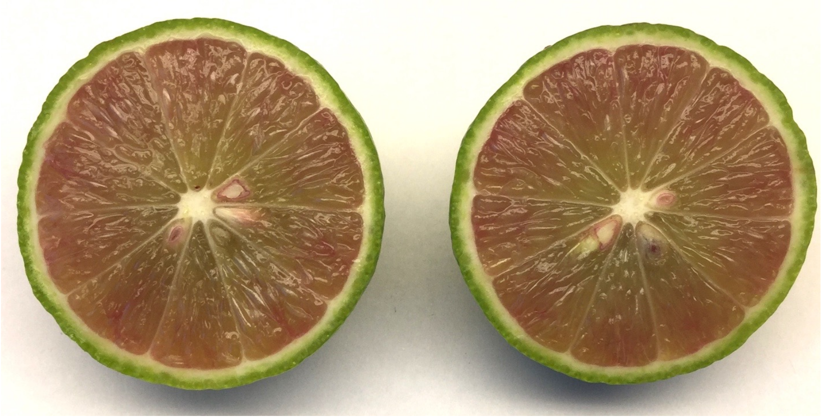

Supplemental Figure S5

Low copy insert. Low expression.
Loss of color as fruit matures.

9-8

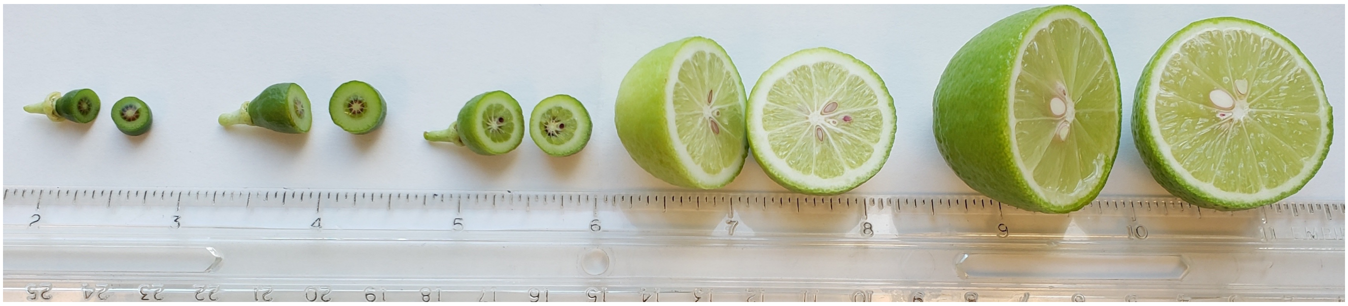


9-31 – not pictured

Supplemental Figure S6

*CitWaxp-Ruby* events of potential commercial interest include those with low copy T-DNA inserts that exhibit substantial anthocyanin accumulation

7-2A

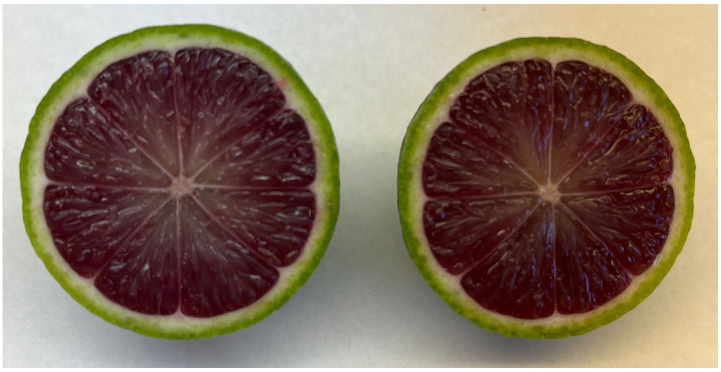

7-10

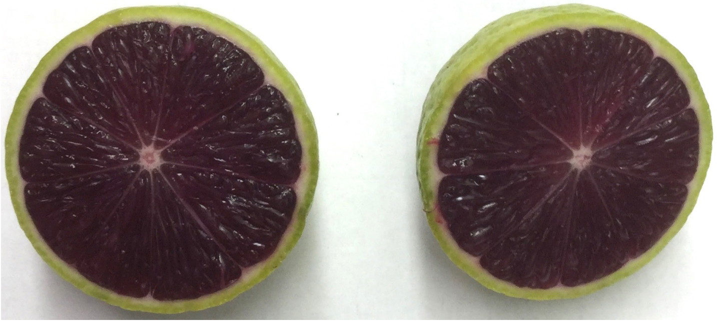

9-1

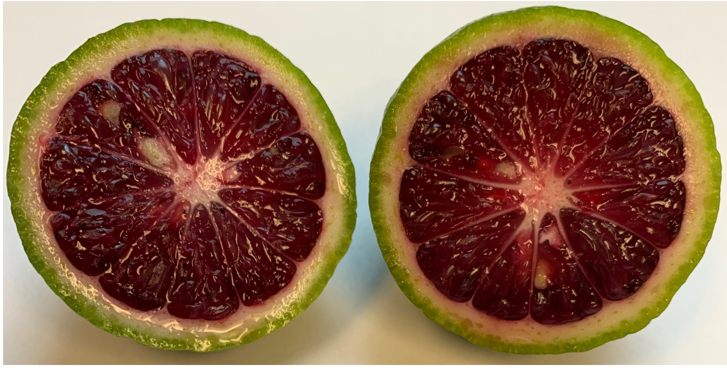

9-10

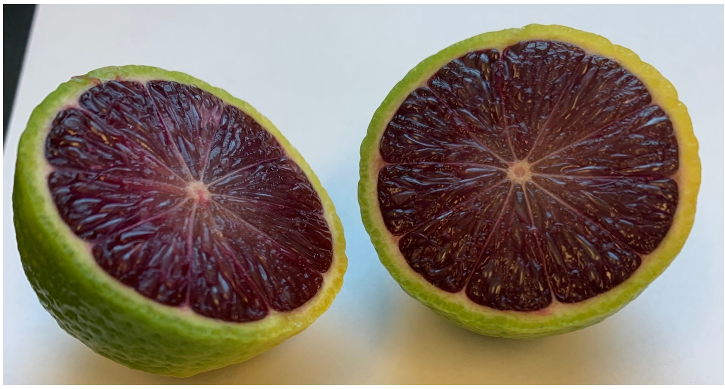

Supplement: Supplementary file 1 [file Data_Sheet_1.PDF]
