# Supplementary material for: Tissue-specific expression of Ruby in Mexican lime (C. aurantifolia) confers anthocyanin accumulation in fruit
Source: Front Plant Sci. 2022 Aug 8;13:945738. doi: 10.3389/fpls.2022.945738 (PMC9393592; doi:10.3389/fpls.2022.945738)
Supplement: Supplementary file 2 [file Data_Sheet_2.PDF]

Supplemental Figure S7 HPLC chromatographic profiles.

Supplemental Figure S7

Moro blood orange control

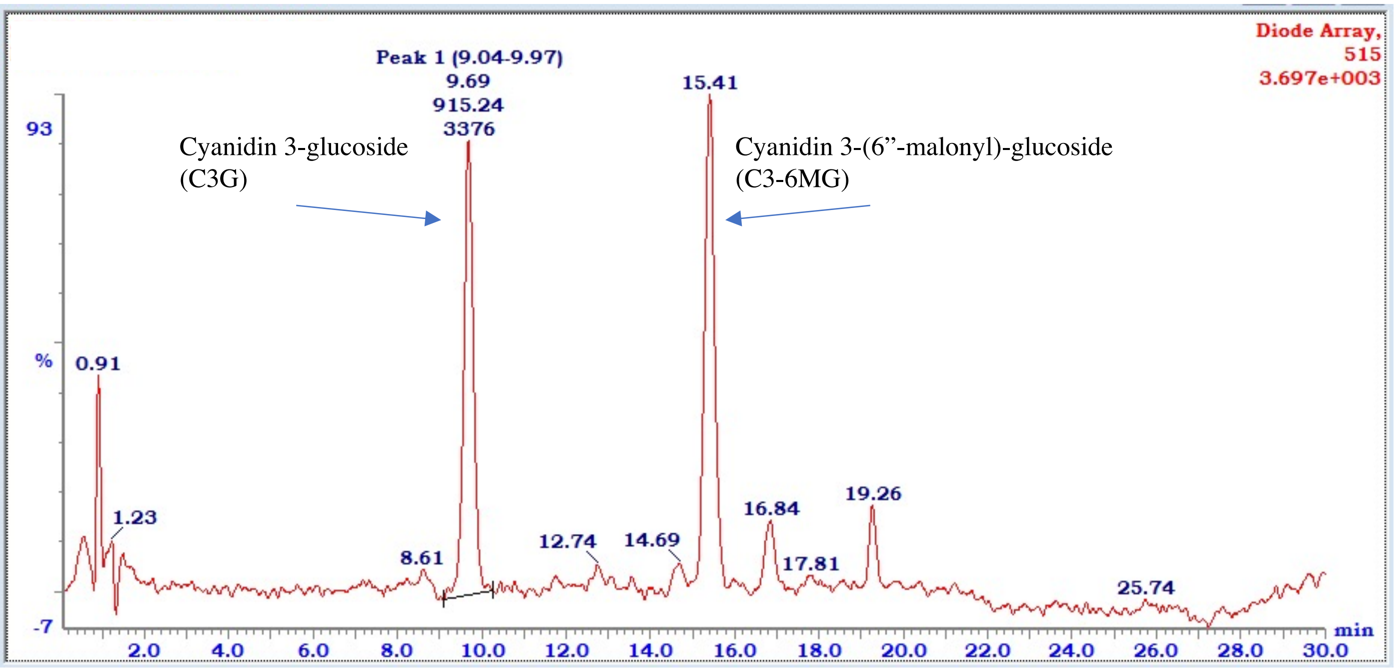

Cyanidin 3-glucoside (C3G) minute 9.7 and cyanidin 3-(6”-malonyl)- β-glucoside (C3-6MG) minute 15.4 observed and previously defined by Lee etal 2002; Scordino et al 2015.

Supplemental Figure S7

Wildtype Mexican lime control

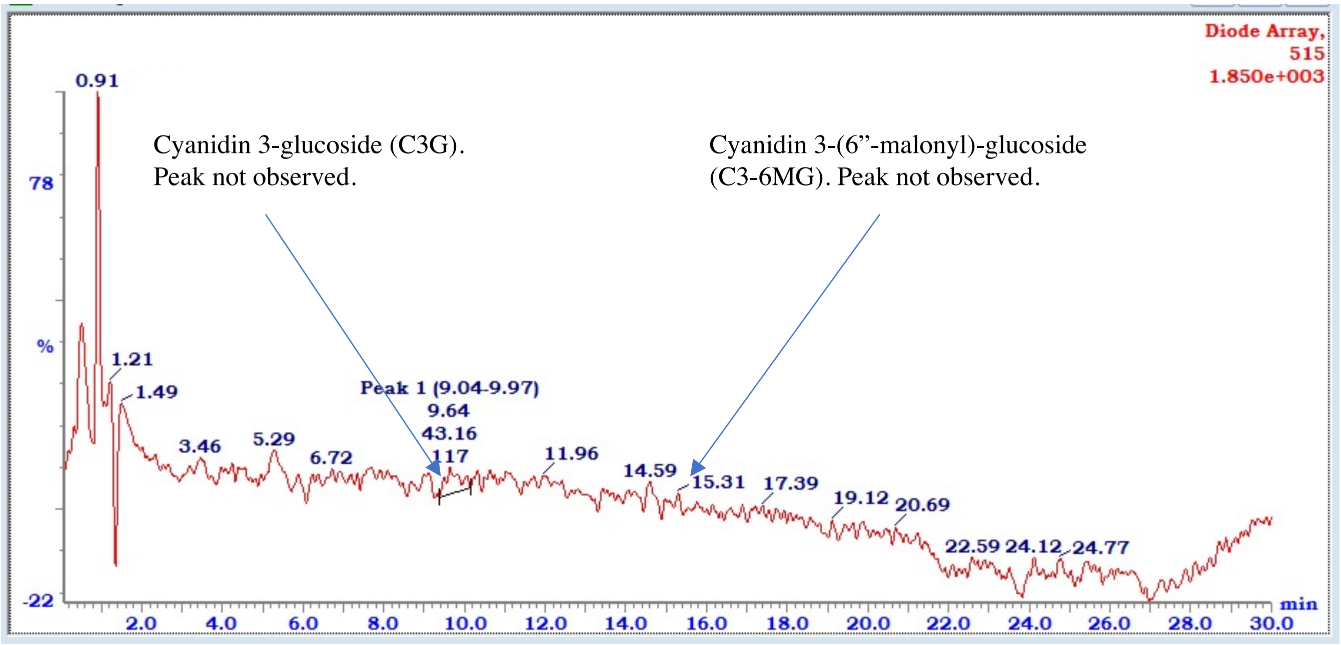

Cyanidin 3-glucoside (C3G) minute 9.7 and cyanidin 3-(6”-malonyl)- β-glucoside (C3-6MG) minute 15.4 not seen over background.

Supplemental Figure S7

CitVO1 1-1

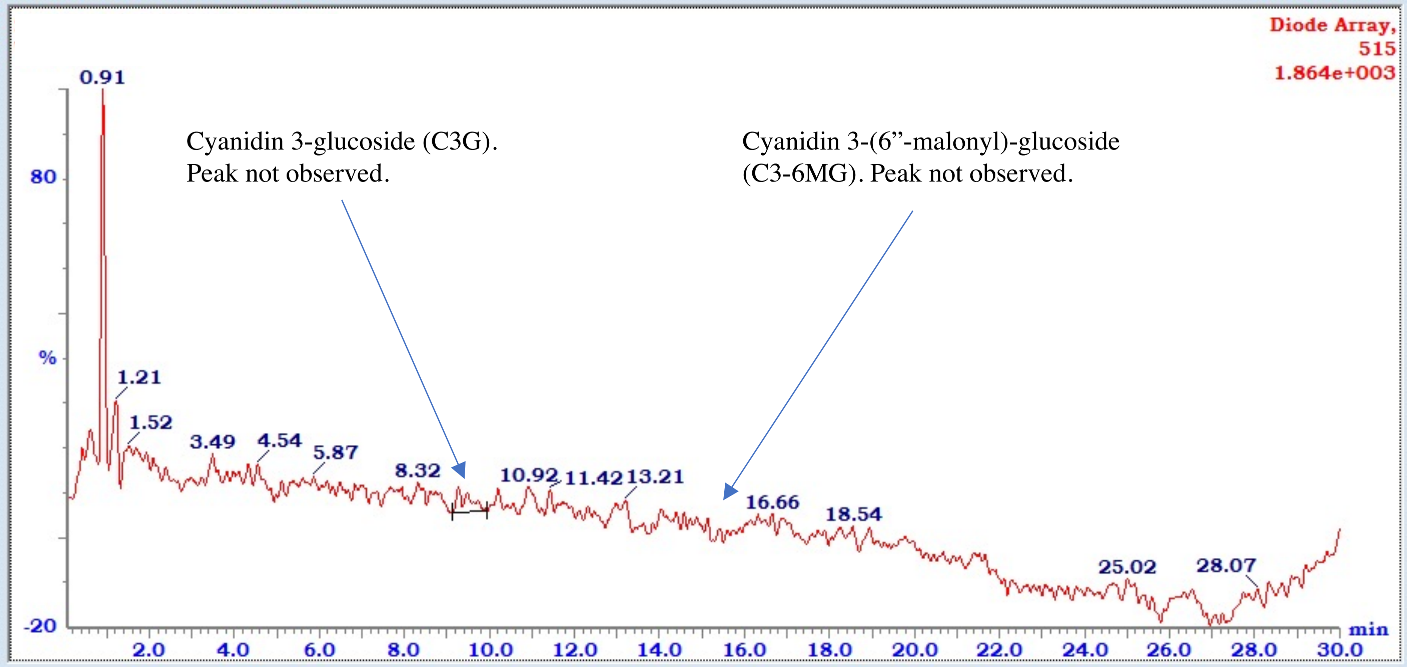

Cyanidin 3-glucoside (C3G) minute 9.7 and cyanidin 3-(6”-malonyl)- β-glucoside (C3-6MG) minute 15.4 not seen over background.

Supplemental Figure S7

CitUNK 4-57

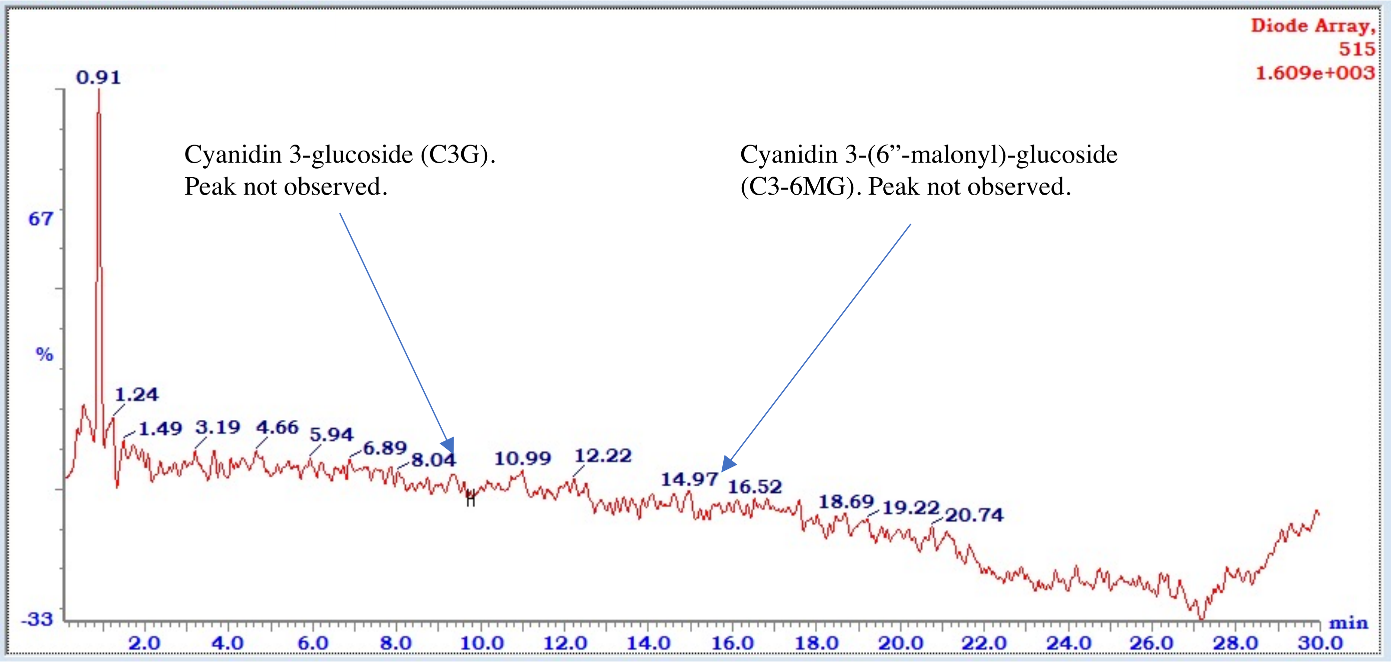

Cyanidin 3-glucoside (C3G) minute 9.7 and cyanidin 3-(6”-malonyl)- β-glucoside (C3-6MG) minute 15.4 not seen over background.

Supplemental Figure S7

PamMybA 5-3A

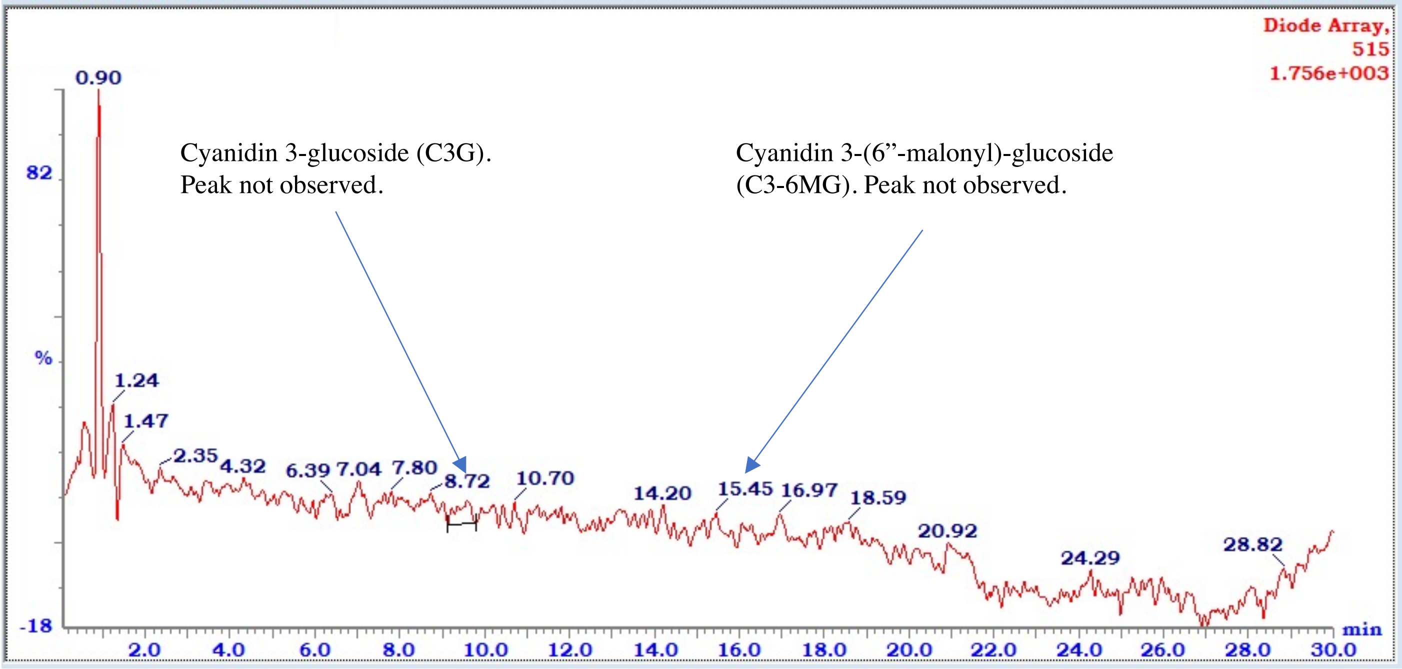

Cyanidin 3-glucoside (C3G) minute 9.7 and cyanidin 3-(6”-malonyl)- β-glucoside (C3-6MG) minute 15.4 not seen over background.

Supplemental Figure S7

SlE8 10-11

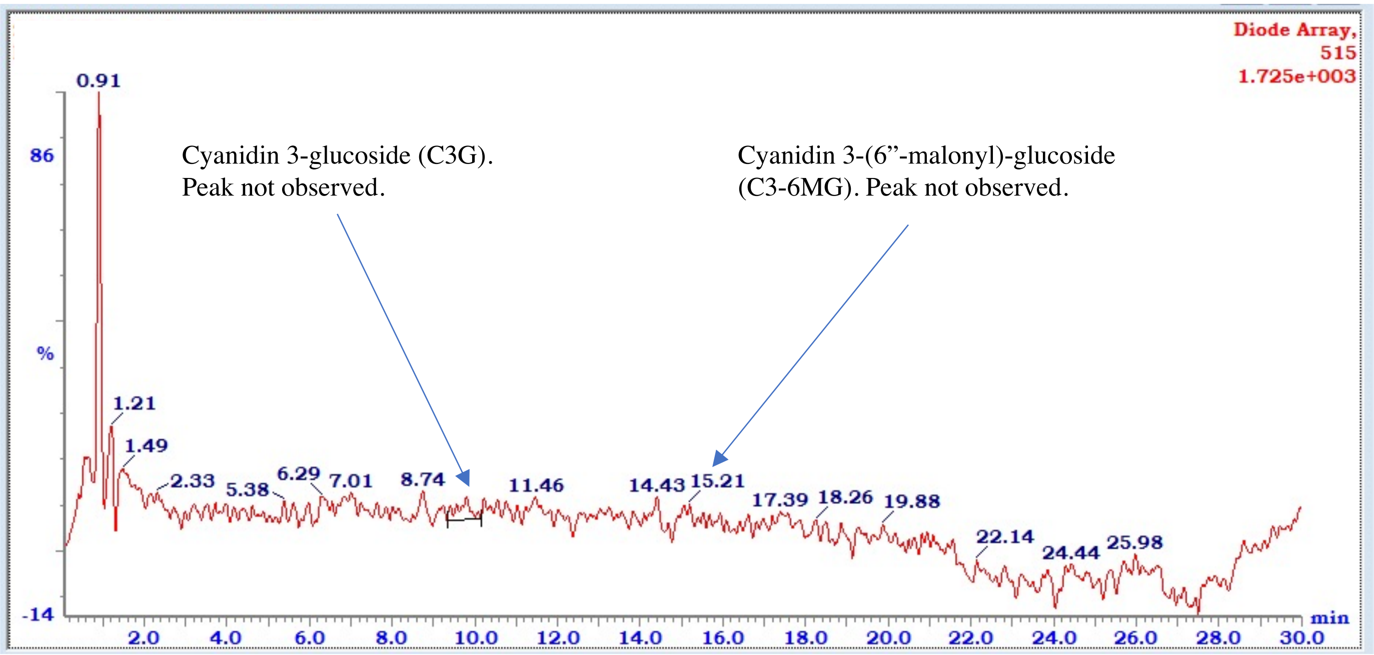

Cyanidin 3-glucoside (C3G) minute 9.7 and cyanidin 3-(6”-malonyl)- β-glucoside (C3-6MG) minute 15.4 not seen over background.

Supplemental Figure S7

CitWax 7-5

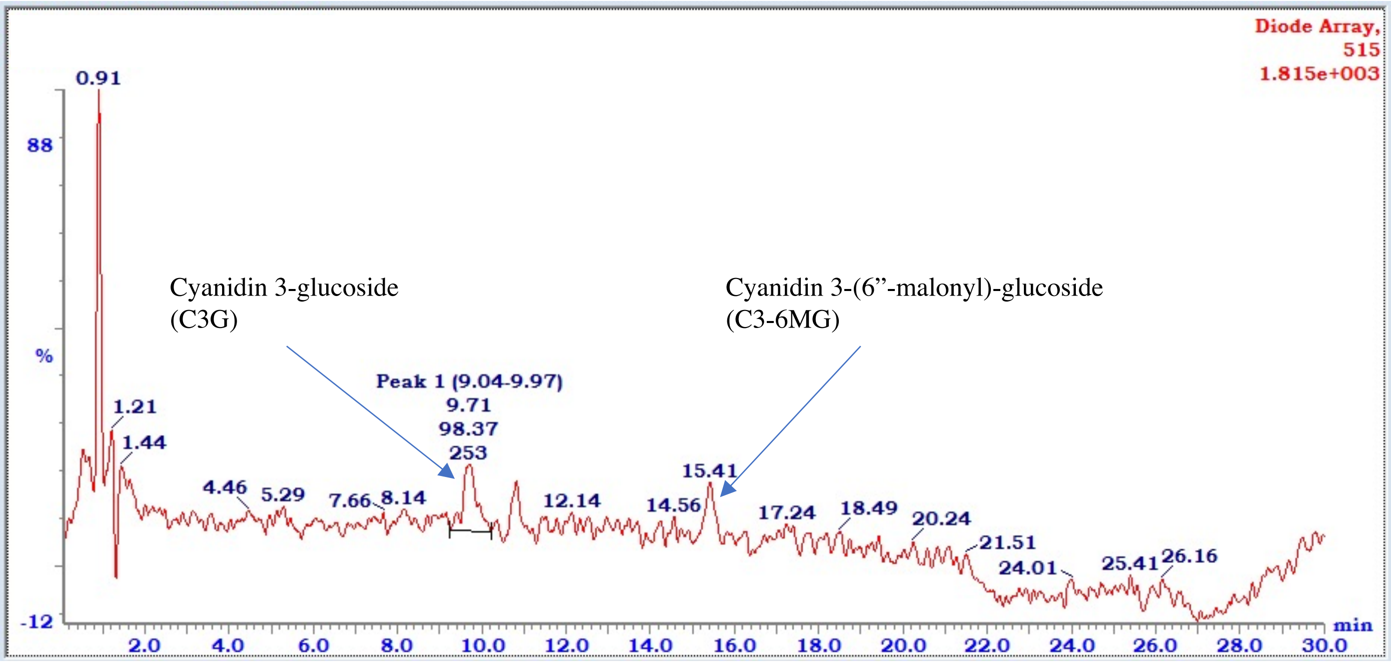

Cyanidin 3-glucoside (C3G) minute 9.7 and cyanidin 3-(6”-malonyl)- β-glucoside (C3-6MG) minute 15.4 observed and previously defined by Lee etal 2002; Scordino et al 2015.

Supplemental Figure S7

CitWax 7-15

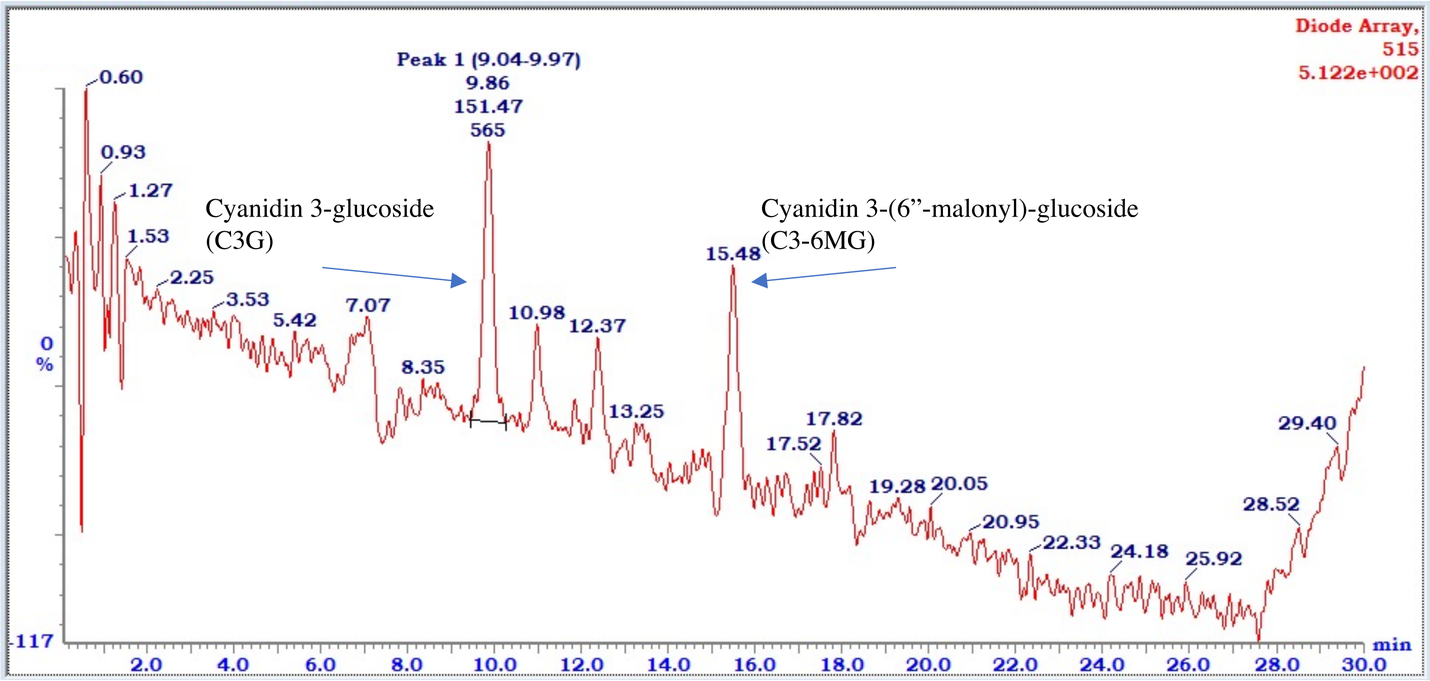

Cyanidin 3-glucoside (C3G) minute 9.7 and cyanidin 3-(6”-malonyl)- β-glucoside (C3-6MG) minute 15.4 observed and previously defined by Lee etal 2002; Scordino et al 2015.

Supplemental Figure S7

CitWax 7-16

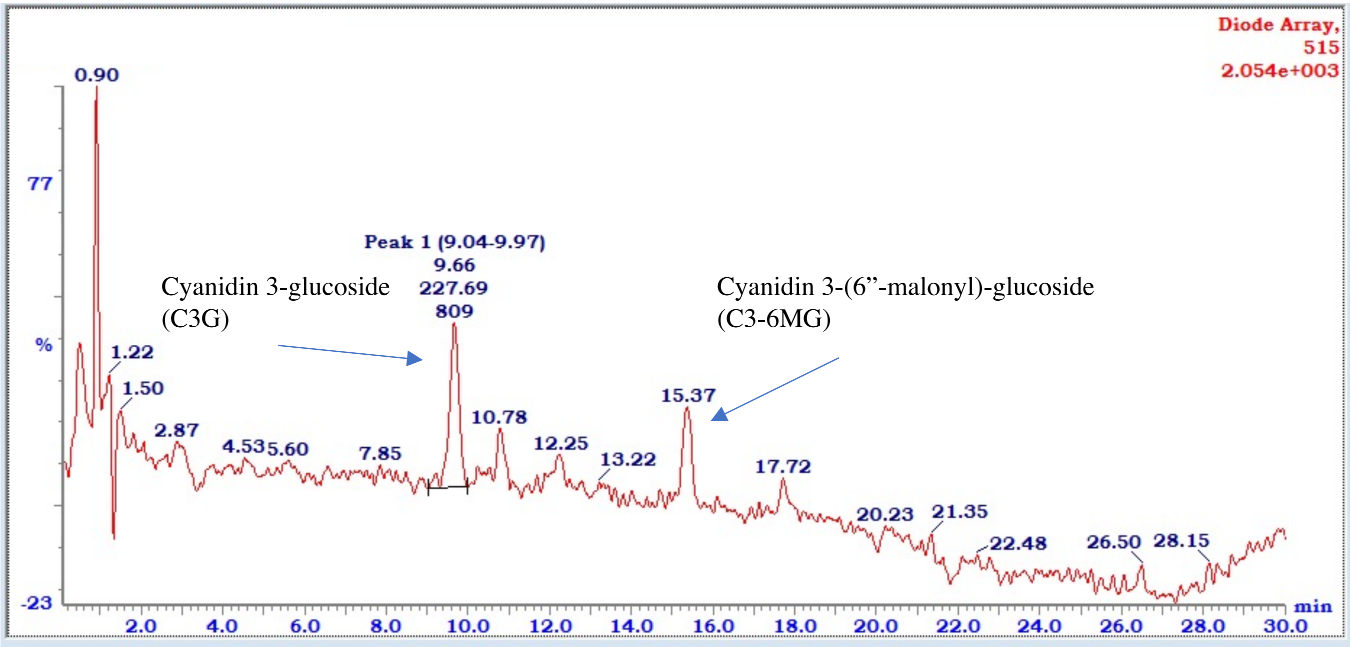

Cyanidin 3-glucoside (C3G) minute 9.7 and cyanidin 3-(6”-malonyl)- β-glucoside (C3-6MG) minute 15.4 observed and previously defined by Lee etal 2002; Scordino et al 2015.

Supplemental Figure S7

CitWax 7-18

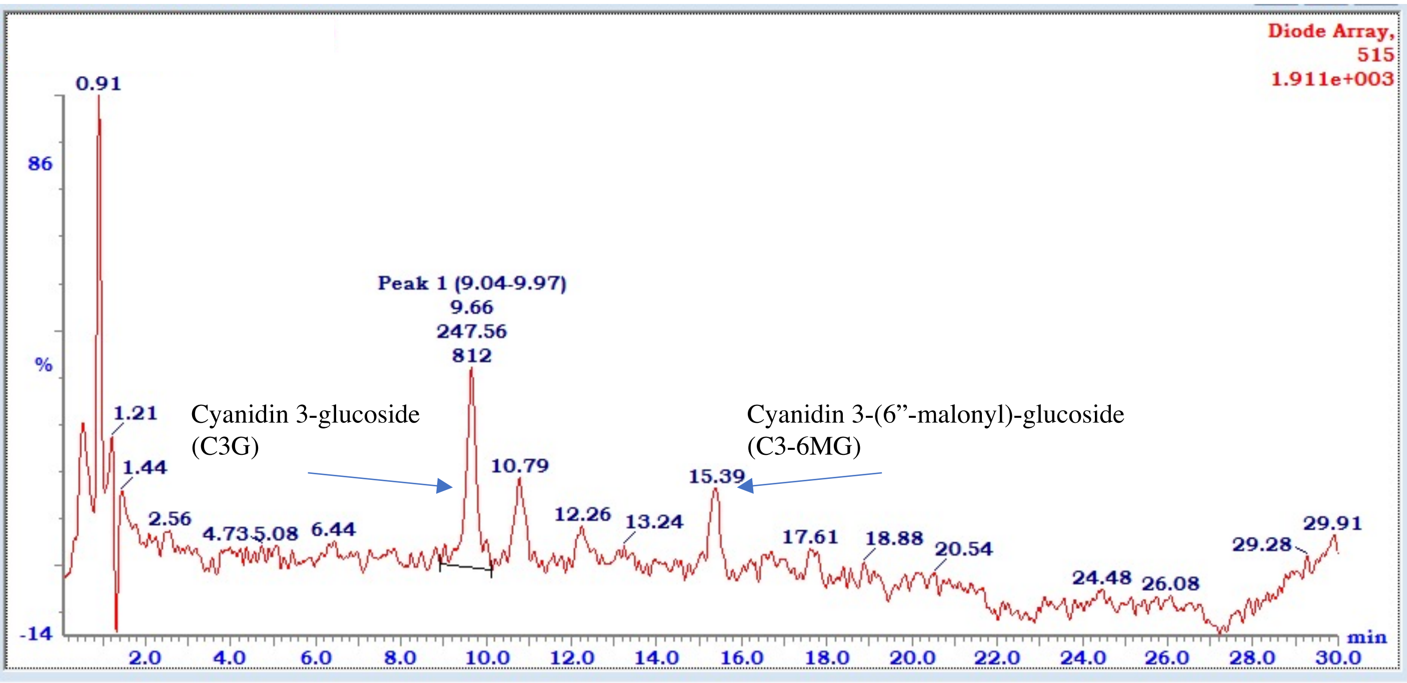

Cyanidin 3-glucoside (C3G) minute 9.7 and cyanidin 3-(6"-malonyl)- β-glucoside (C3-6MG) minute 15.4 observed and previously defined by Lee etal 2002; Scordino et al 2015.

Supplemental Figure S7

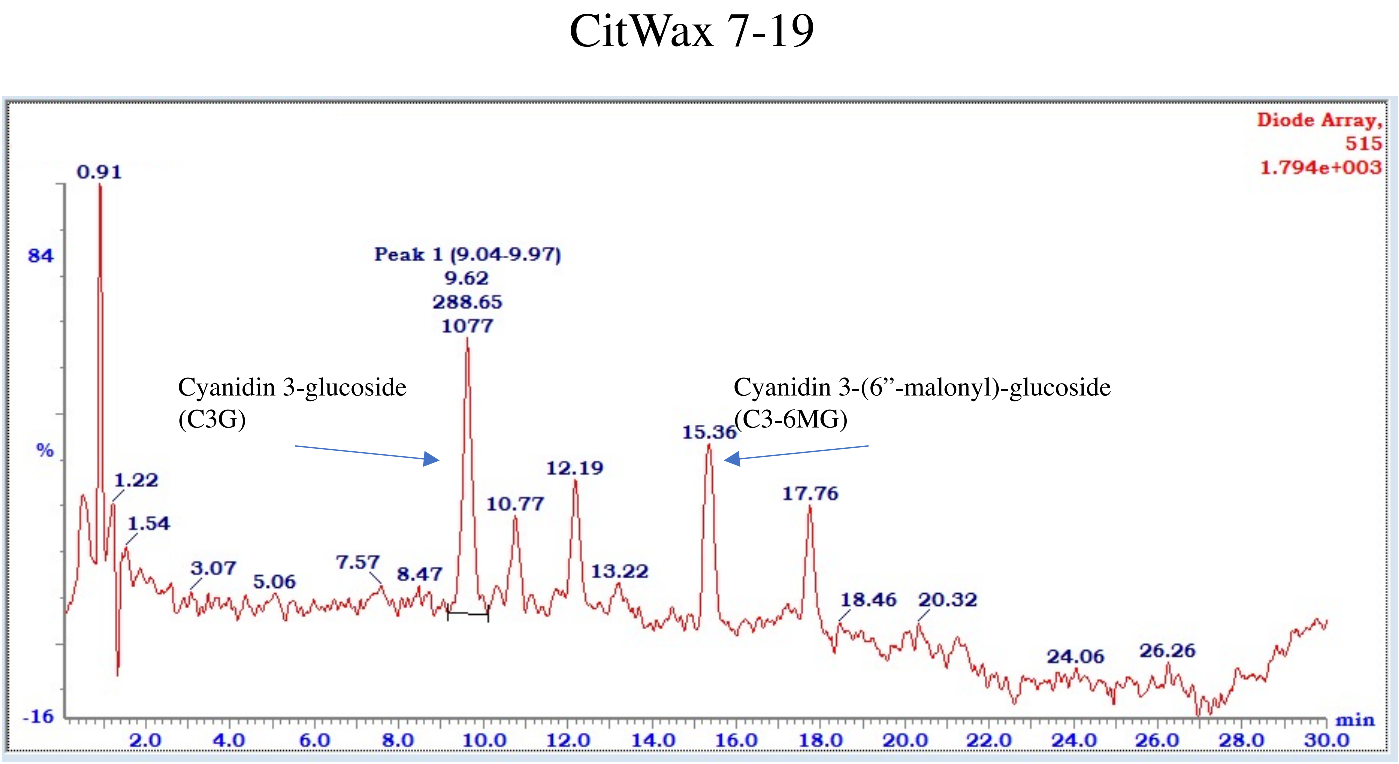

Cyanidin 3-glucoside (C3G) minute 9.7 and cyanidin 3-(6”-malonyl)- β-glucoside (C3-6MG) minute 15.4 observed and previously defined by Lee etal 2002; Scordino et al 2015.

Supplemental Figure S7

CitWax 9-1

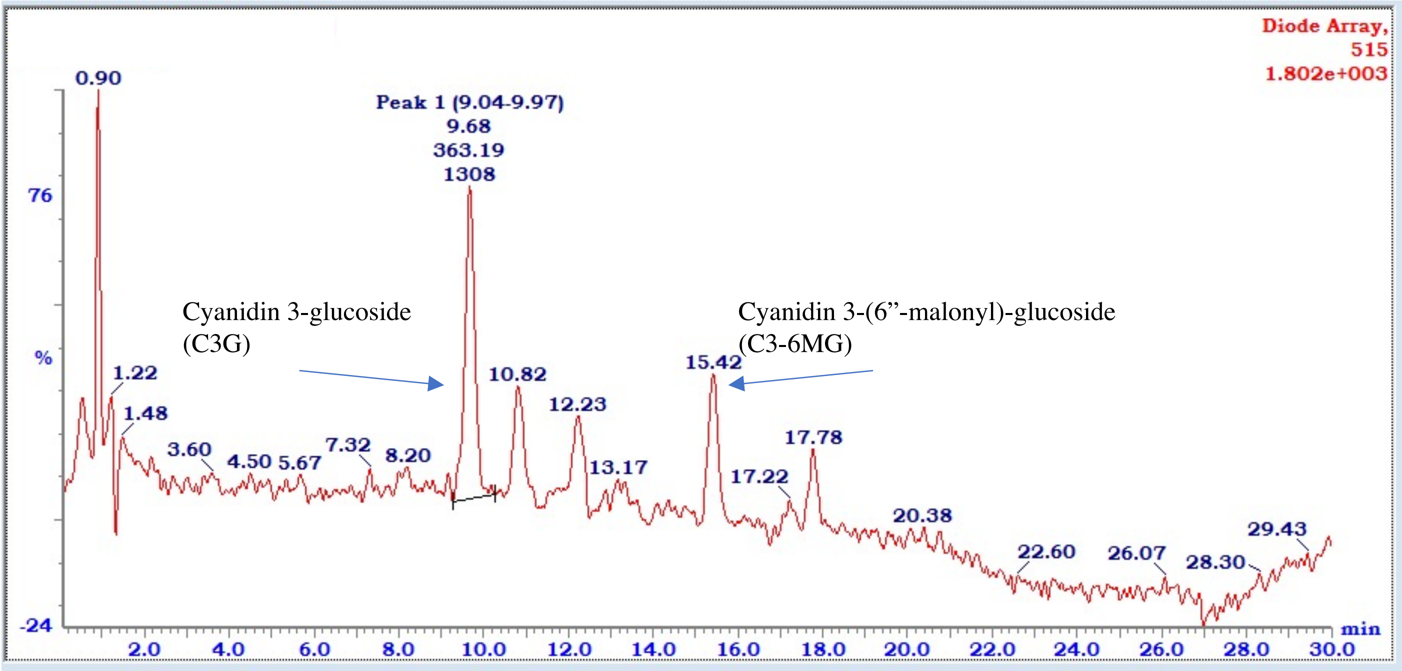

Cyanidin 3-glucoside (C3G) minute 9.7 and cyanidin 3-(6"-malonyl)- β-glucoside (C3-6MG) minute 15.4 observed and previously defined by Lee etal 2002; Scordino et al 2015.

Supplemental Figure S7

CitWax 9-9

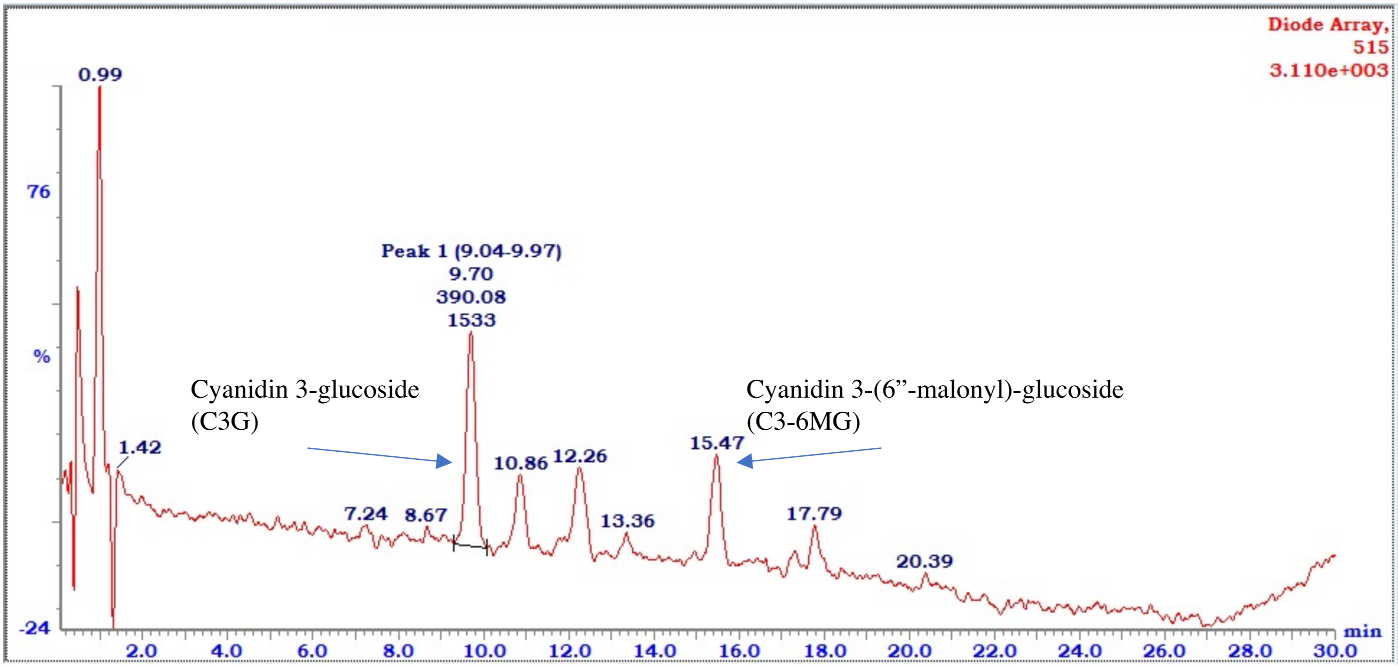

Cyanidin 3-glucoside (C3G) minute 9.7 and cyanidin 3-(6”-malonyl)- β-glucoside (C3-6MG) minute 15.4 observed and previously defined by Lee etal 2002; Scordino et al 2015.

Supplemental Figure S7

CitWax 9-12

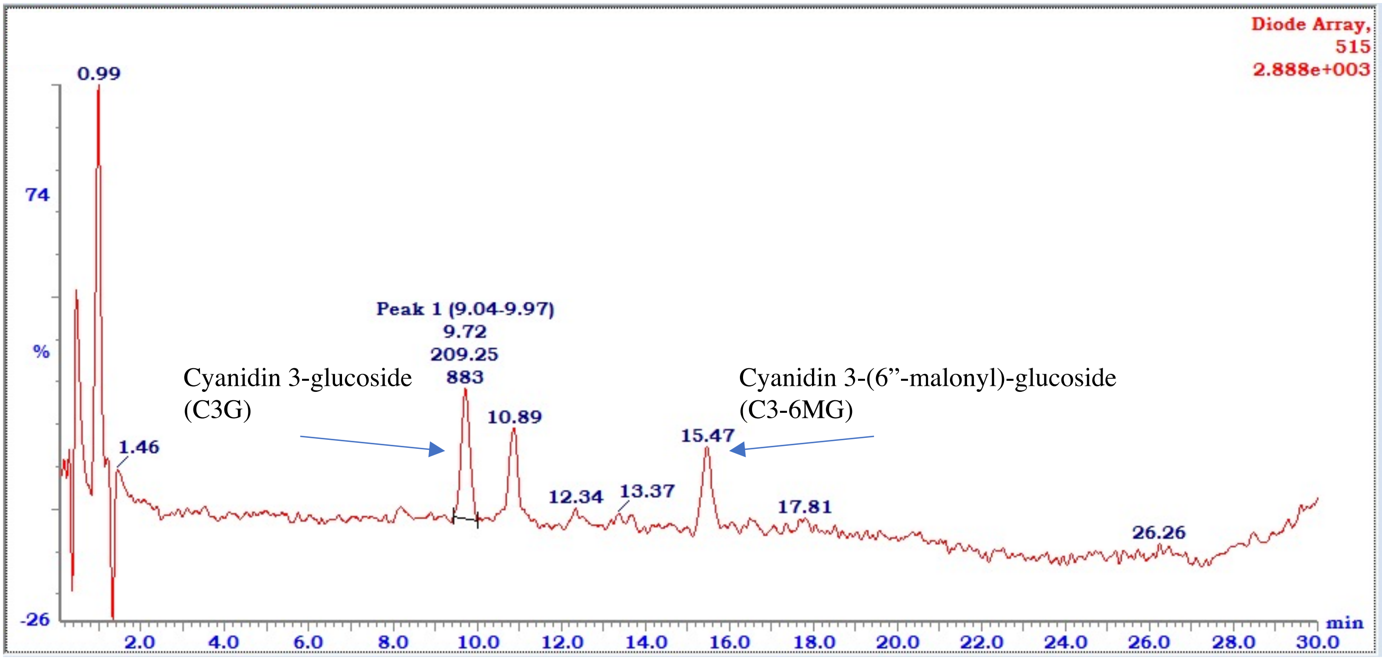

Cyanidin 3-glucoside (C3G) minute 9.7 and cyanidin 3-(6"-malonyl)- β-glucoside (C3-6MG) minute 15.4 observed and previously defined by Lee etal 2002; Scordino et al 2015.

Supplemental Figure S7

CitWax 9-14

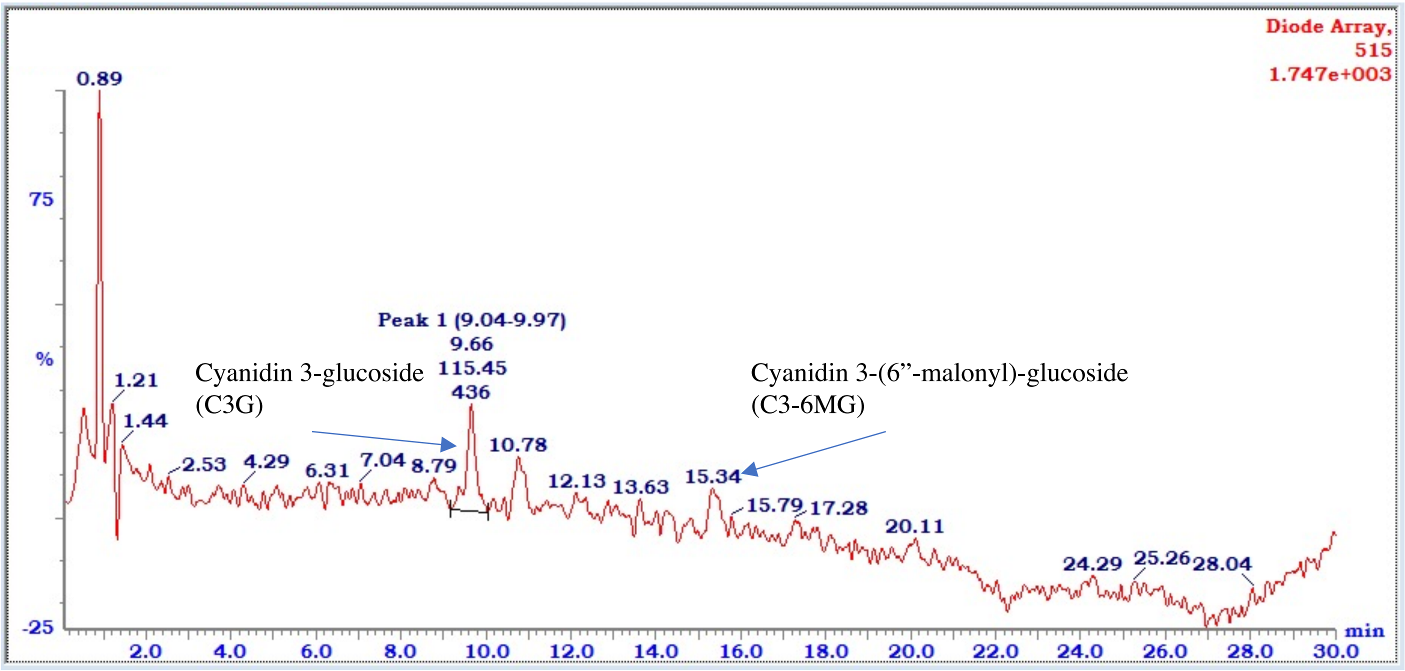

Cyanidin 3-glucoside (C3G) minute 9.7 and cyanidin 3-(6”-malonyl)- β-glucoside (C3-6MG) minute 15.4 observed and previously defined by Lee etal 2002; Scordino et al 2015.

Supplemental Figure S7

CitWax 9-23

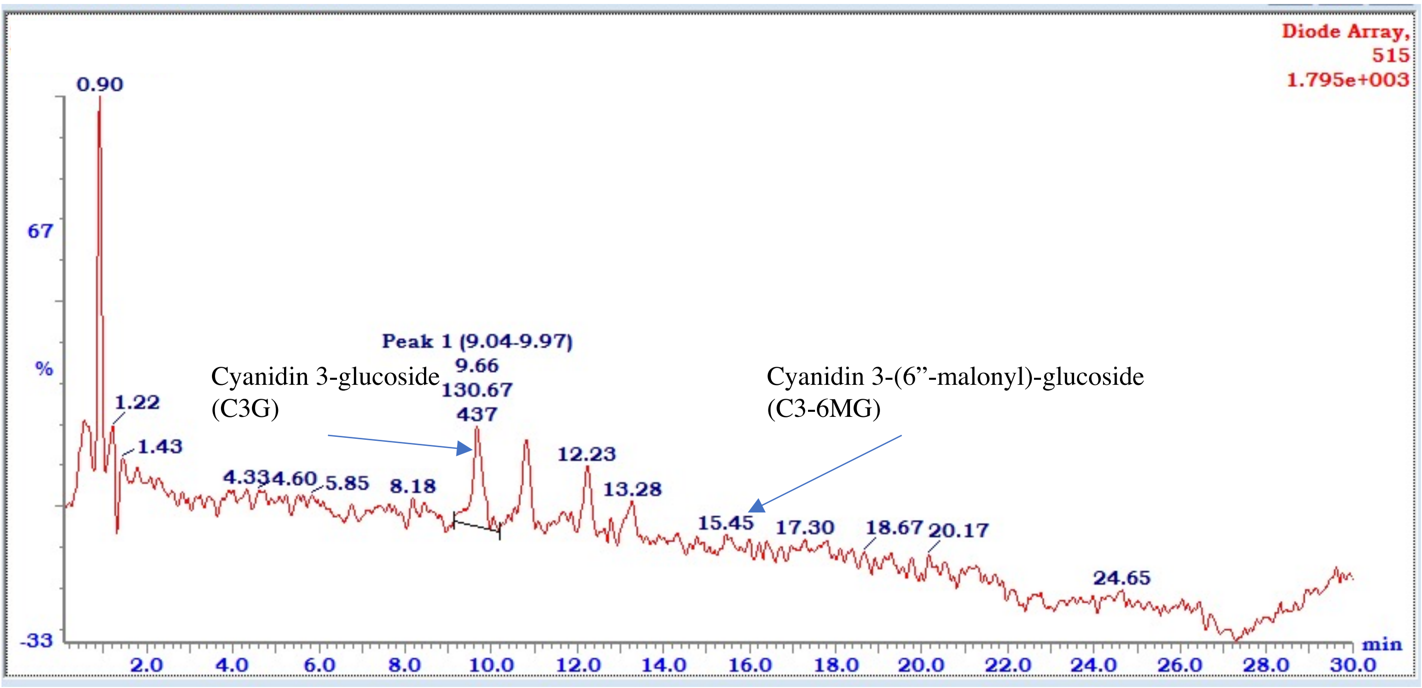

Cyanidin 3-glucoside (C3G) minute 9.7 and cyanidin 3-(6”-malonyl)- β-glucoside (C3-6MG) minute 15.4 observed and previously defined by Lee etal 2002; Scordino et al 2015.

Supplemental Figure S7

CitWax 9-24

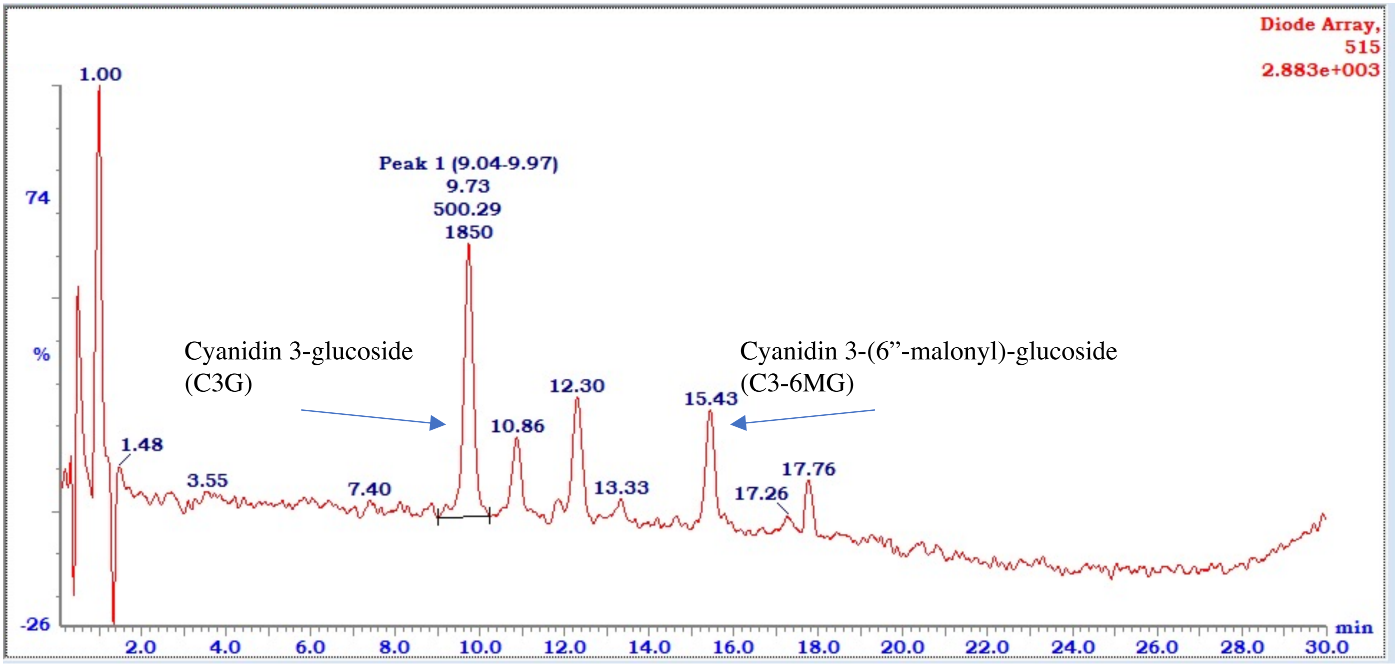

Cyanidin 3-glucoside (C3G) minute 9.7 and cyanidin 3-(6”-malonyl)- β-glucoside (C3-6MG) minute 15.4 observed and previously defined by Lee etal 2002; Scordino et al 2015.

Supplemental Figure S8
MS and MS-MS profiles.

Supplemental Figure S8

Blood Orange
MS1

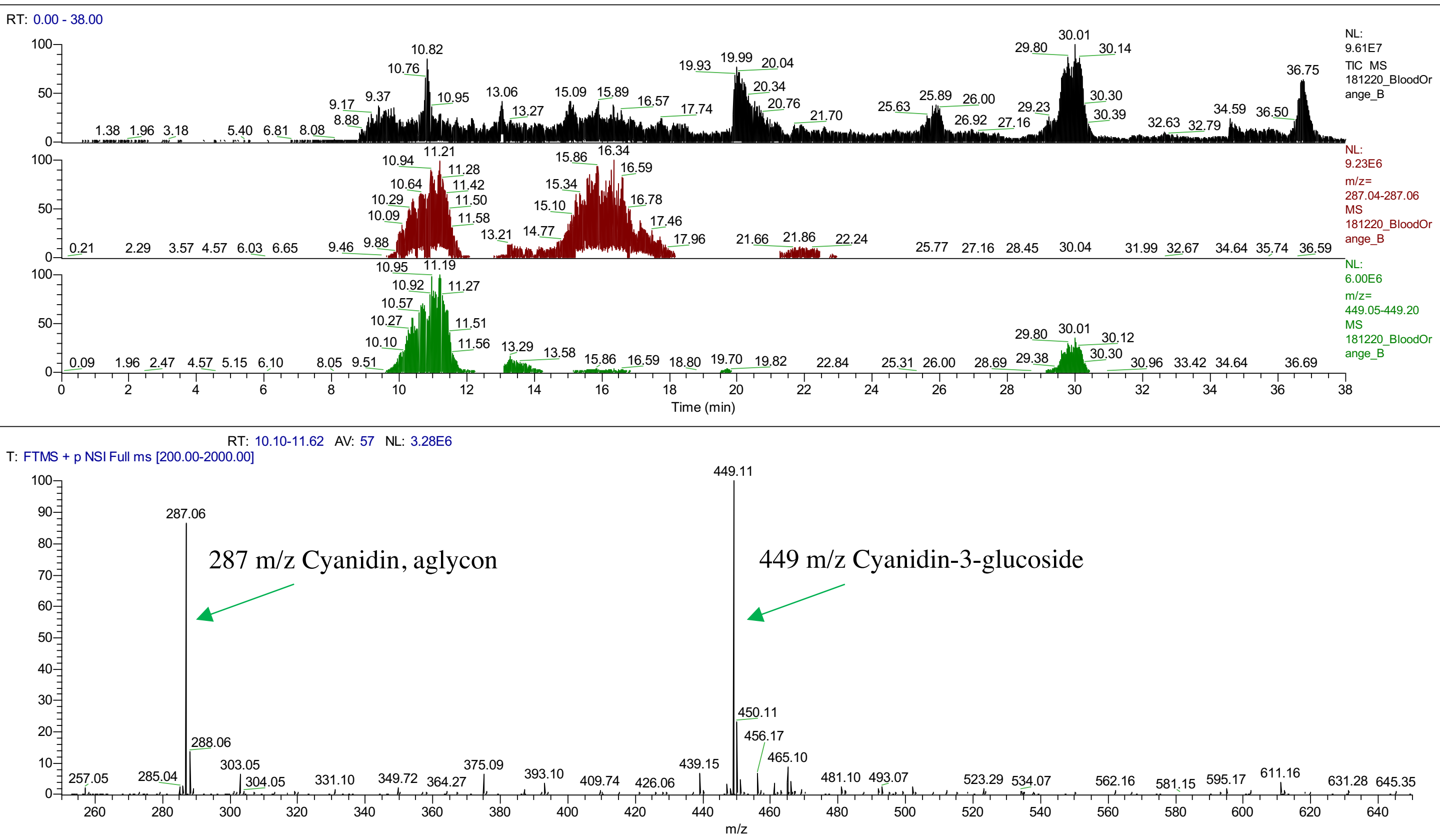

Supplemental Figure 8A. Blood orange juice MS1 analysis. Top panel shows the ion capture of all charge in compounds from MS survey scan range of 200-2000 m/z . Second panel shows specific ion capture from 287.04 to 287.06 m/z. Third panel shown specific ion capture from 449.05 to 449.20 m/z. Forth panel shows the strongest specific peaks from the full MS survey scan range of 200-2000 m/z at the retention times 10.10-11.62 minutes.

Supplemental Figure S8

Blood Orange
MS2

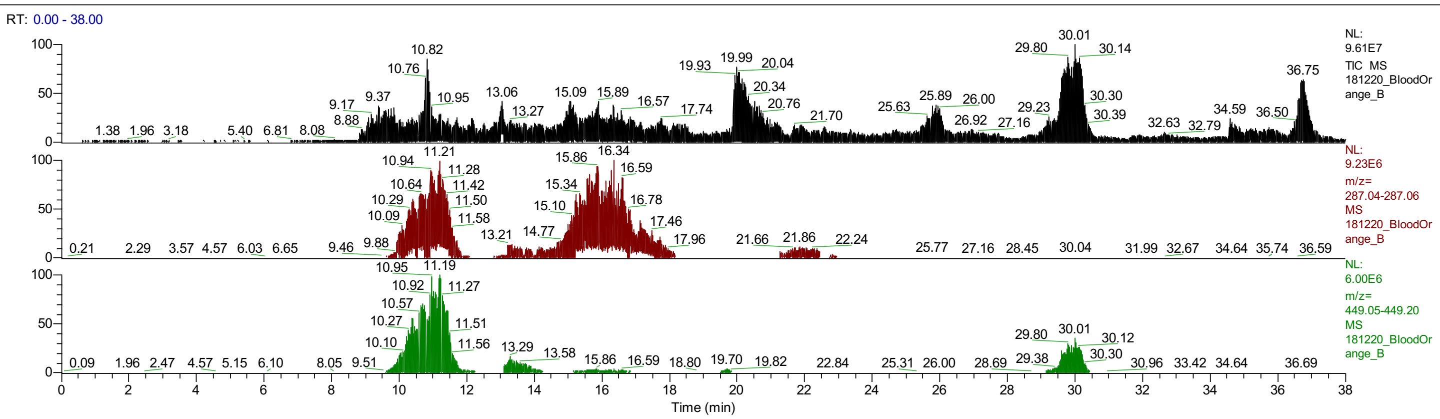

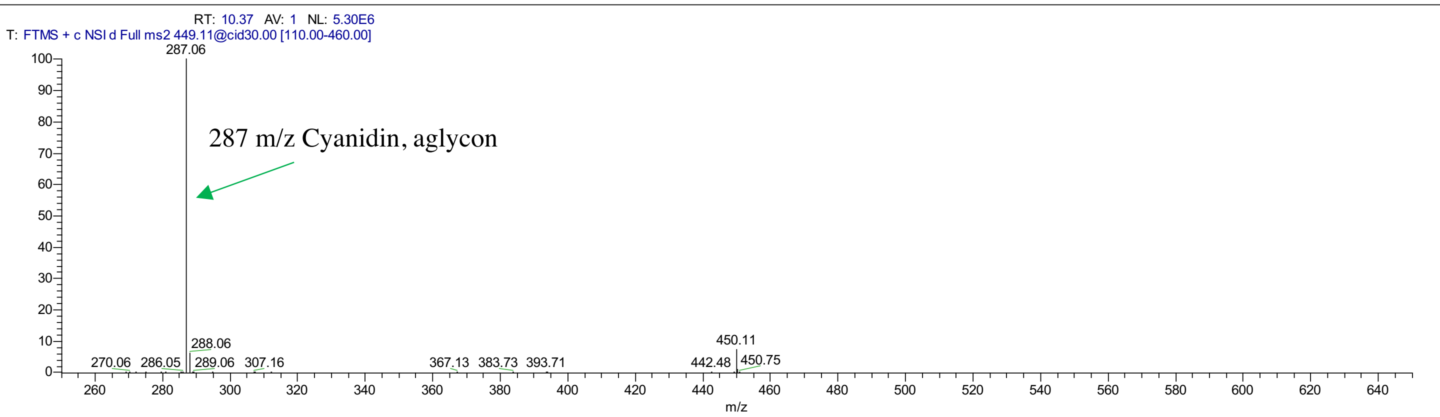

Supplemental Figure 8B. Blood orange juice MS2 analysis. Top panel shows the ion capture of all charge in compounds from MS survey scan range of 200-2000 m/z . Second panel shows specific ion capture from 287.04 to 287.06 m/z. Third panel shown specific ion capture from 449.05 to 449.20 m/z. Forth panel shows the strongest peaks after electrospray fragmentation from the full MS survey scan range of 449.11 m/z at the retention times 10.37 minutes.

Supplemental Figure S8

Mexican Lime wild-type
MS1

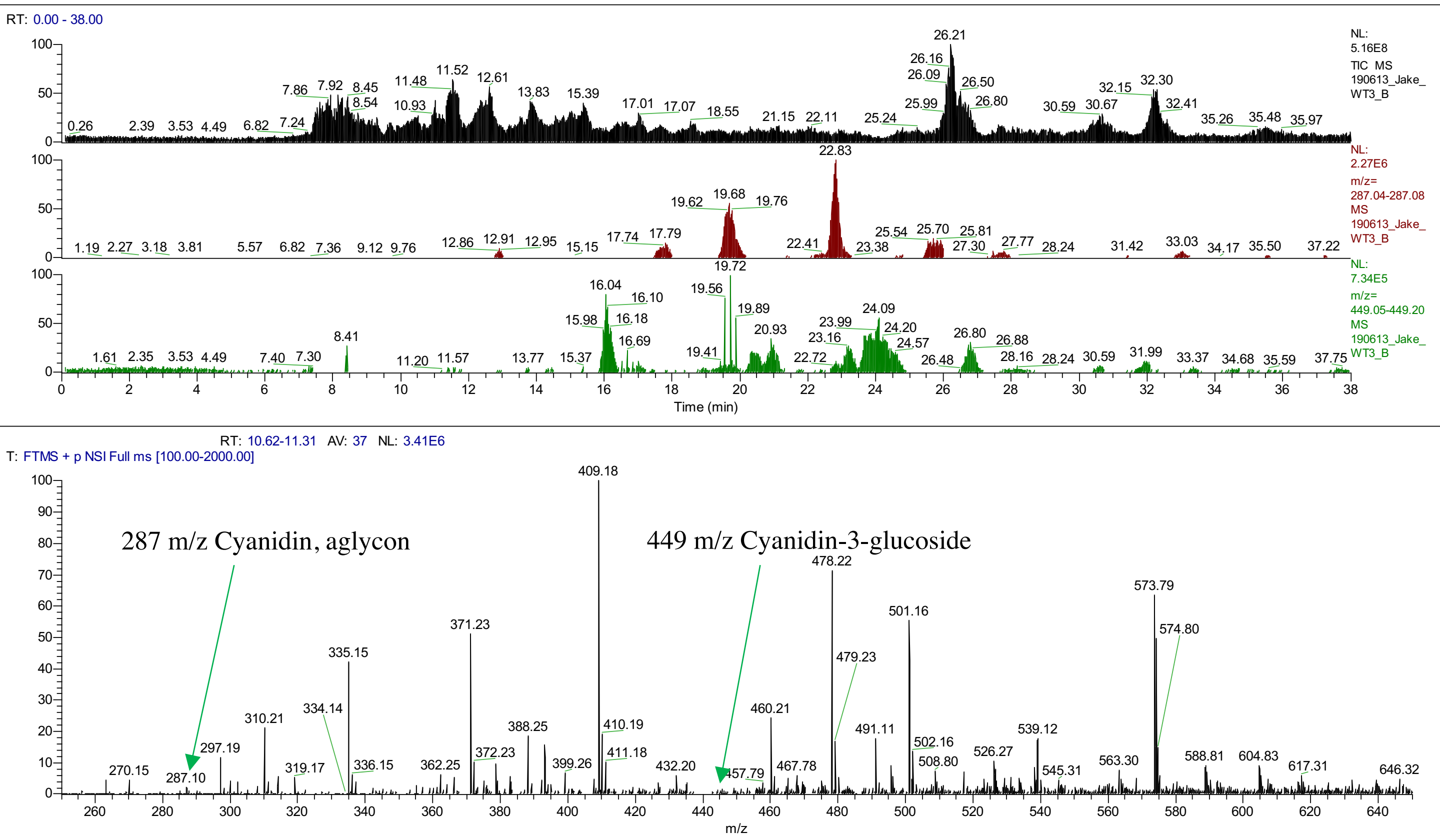

Supplemental Figure 8C. Mexican lime wild-type juice MS1 analysis. Top panel shows the ion capture of all charge in compounds from MS survey scan range of 200-2000 m/z . Second panel shows specific ion capture from 287.04 to 287.06 m/z. Third panel shown specific ion capture from 449.05 to 449.20 m/z. Forth panel shows the strongest specific peaks from the full MS survey scan range of 200-2000 m/z at the retention times 10.10-11.62 minutes. As peaks were not observed at the 449 m/z (cyanidin 3-glucoside (C3G)), MS2 was not conducted.

Supplemental Figure S8

CitWax 7-19
MS1

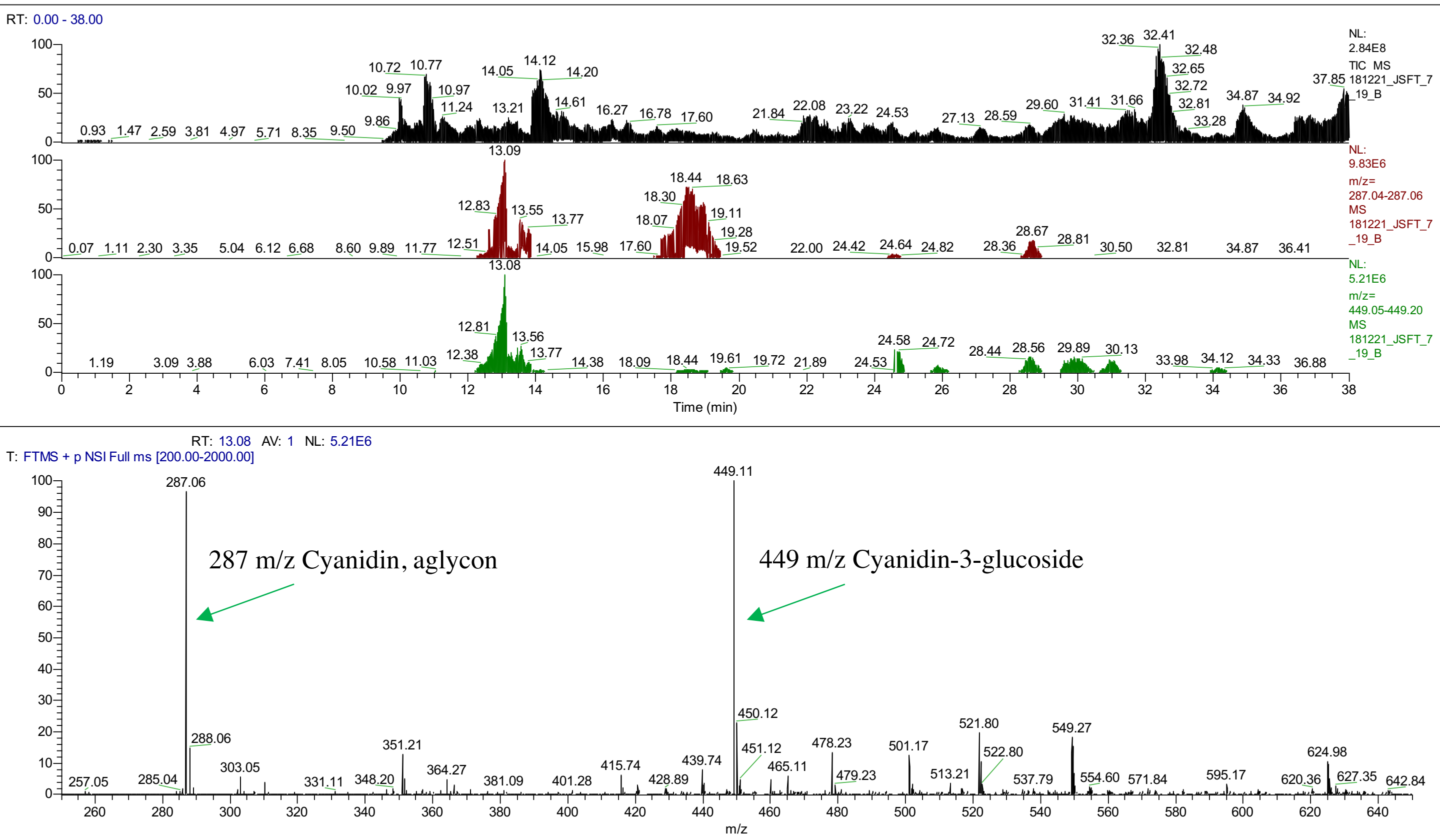

Supplemental Figure 8D. CitWax 7-19 juice MS1 analysis. Top panel shows the ion capture of all charge in compounds from MS survey scan range of 200-2000 m/z . Second panel shows specific ion capture from 287.04 to 287.06 m/z. Third panel shown specific ion capture from 449.05 to 449.20 m/z. Forth panel shows the strongest specific peaks from the full MS survey scan range of 200-2000 m/z at the retention time of 13.08 minutes.

Supplemental Figure S8

CitWax 7-19
MS2

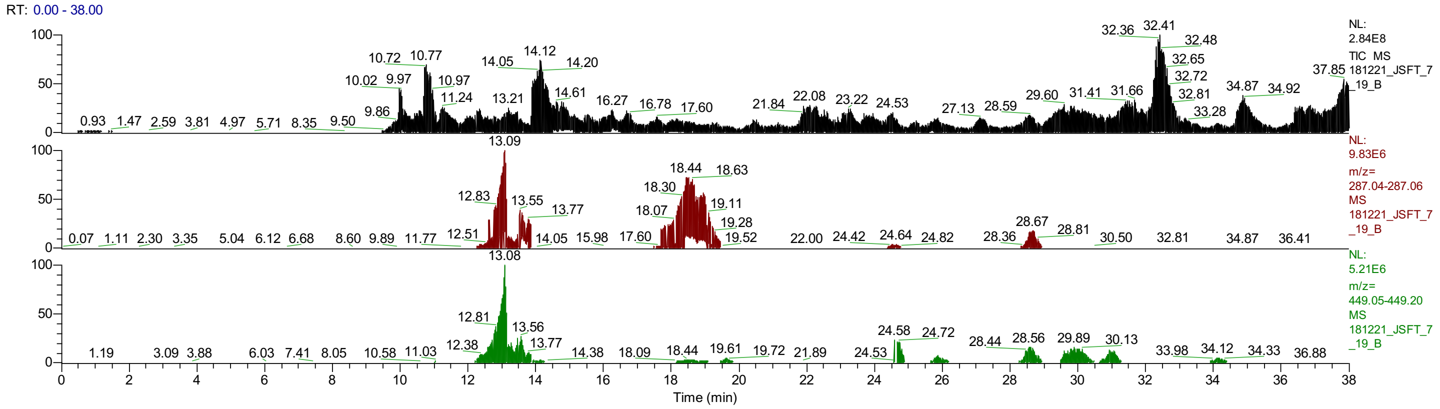

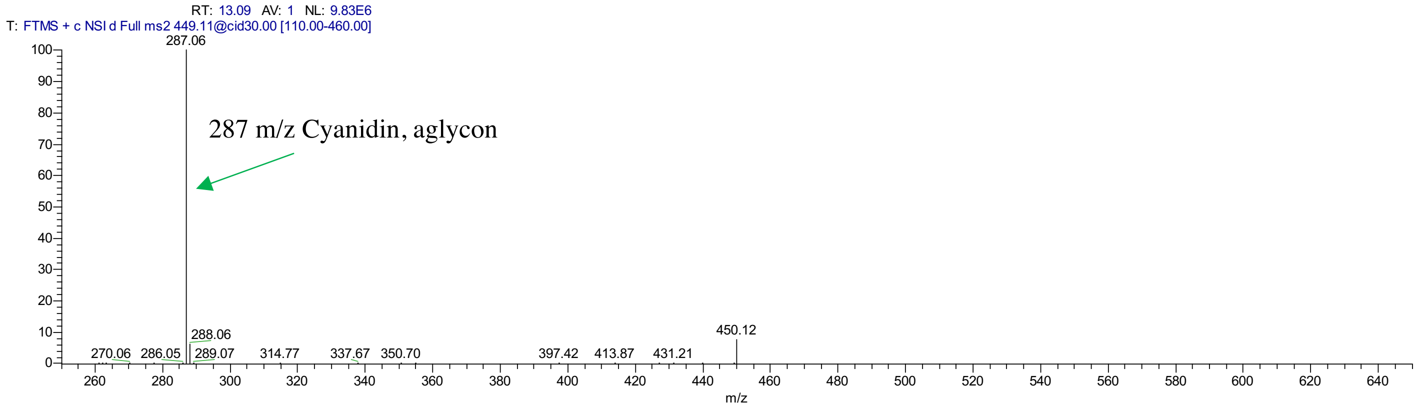

Supplemental Figure 8E. CitWax 7-19 juice MS2 analysis. Top panel shows the ion capture of all charge in compounds from MS survey scan range of 200-2000 m/z . Second panel shows specific ion capture from 287.04 to 287.06 m/z. Third panel shown specific ion capture from 449.05 to 449.20 m/z. Forth panel shows the strongest peaks after electrospray fragmentation from the full MS survey scan range of 449.11 m/z at the retention times 13.09 minutes.

Supplemental Figure S8

CitWax 7-19
MS1/MS2

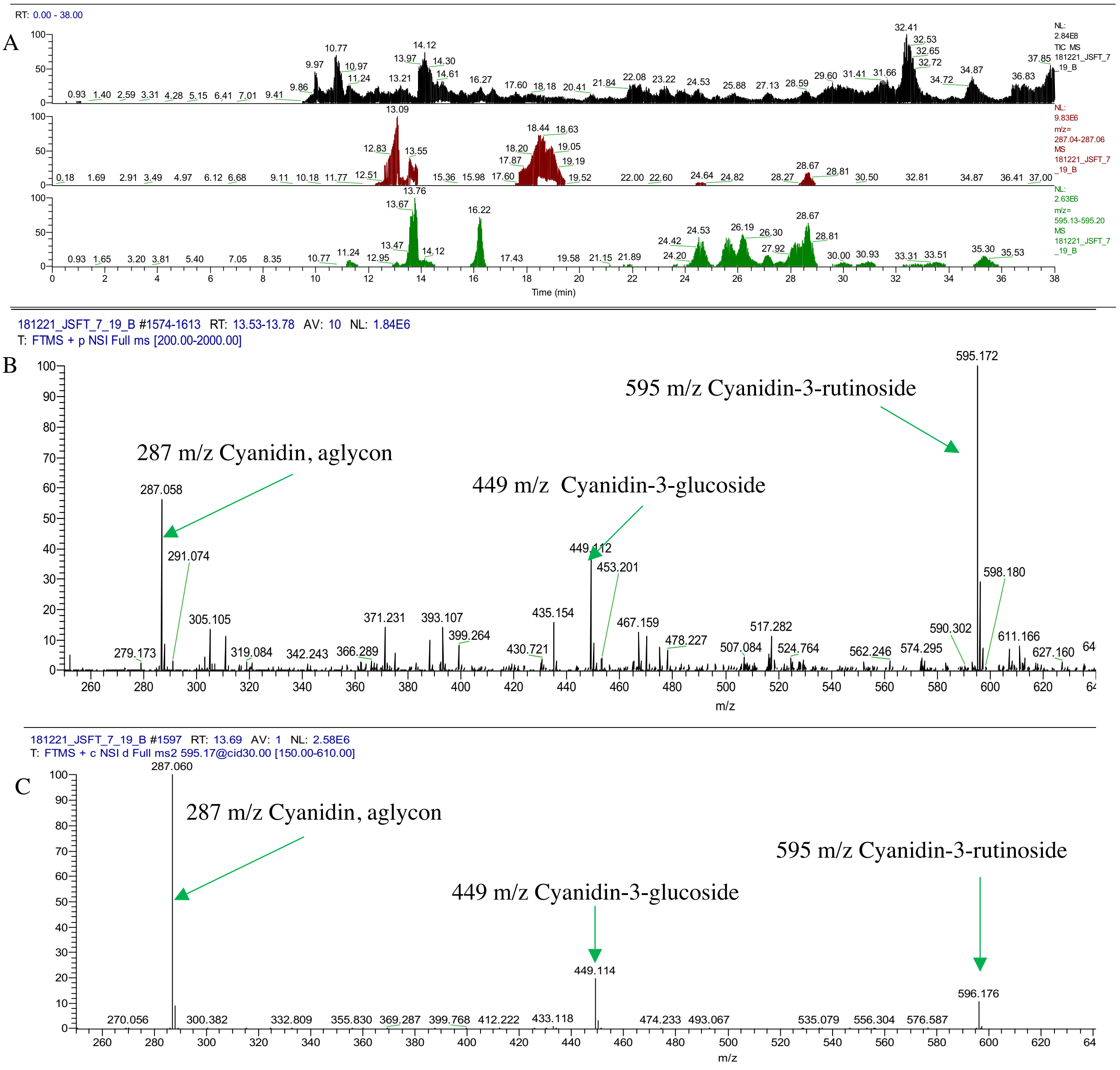

Supplemental Figure 8E. CitWax 7-19 juice MS2 analysis. Mass Spectral Analysis (HPLC-MS-MS) profiles of fresh squeezed juice of line Citwax 7-19. Cyanidin 3-glucoside (C3G), cyanidin 3-(6"-malonyl)-glucoside (C3-6MG) and cyanidin 3-rutinoside (C3R). Values are peak heights based on mass spectral counts and relative to signal intensity measured by the detector. A) HPLC B) Full MS at retention time 13.58-13.78. Mass scan range was 200-2000 m/z. C) MS2 at retention time 13.69. Mass scan range was 150-610 m/z. The MS-MS spectra was determined on the basis of the fragmentation of the ions at 595 m/z.

Supplemental Figure S8

CitWax 9-9
MS1

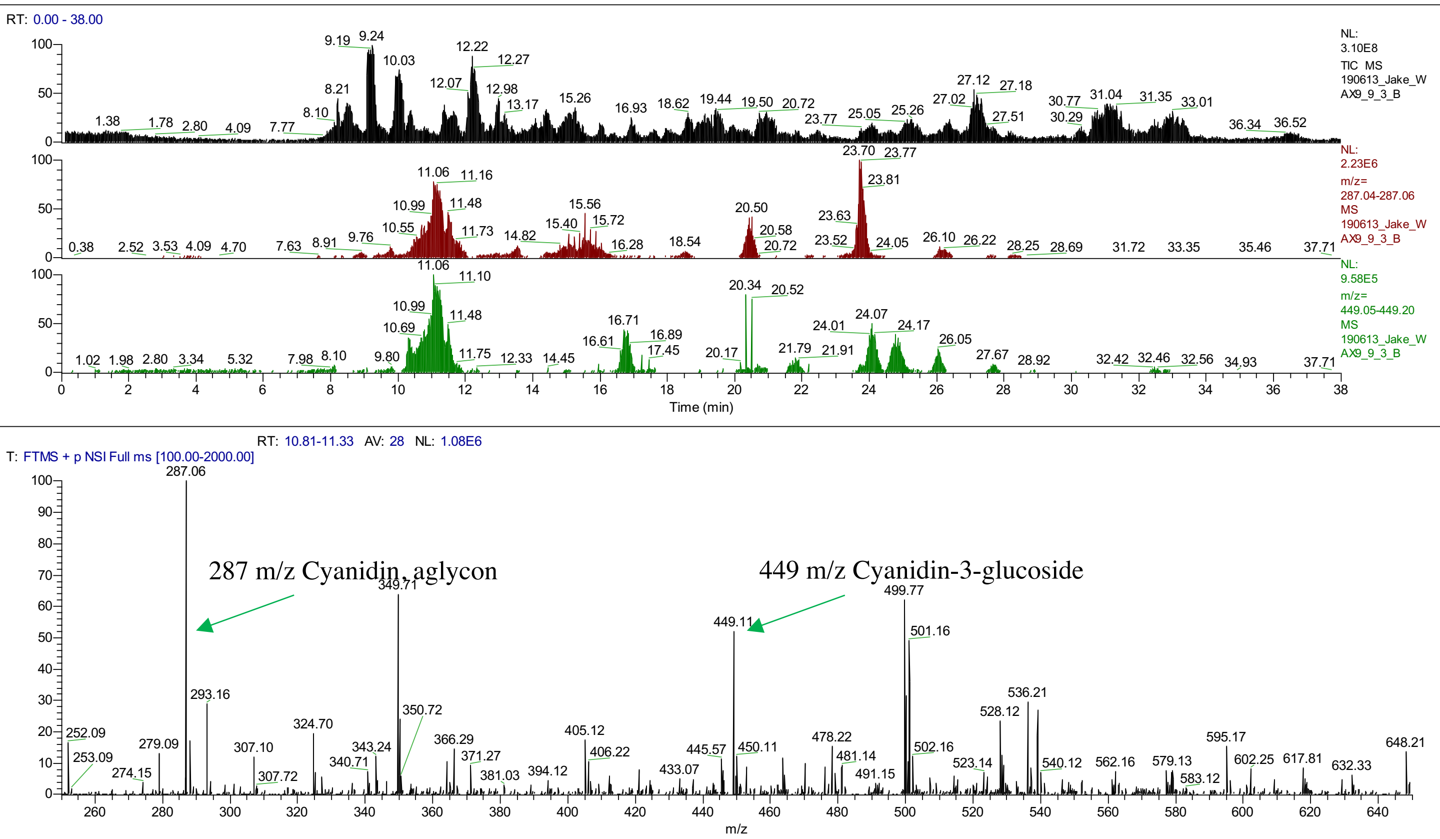

Supplemental Figure 8F. CitWax 9-9 juice MS1 analysis. Top panel shows the ion capture of all charge in compounds from MS survey scan range of 200-2000 m/z . Second panel shows specific ion capture from 287.04 to 287.06 m/z. Third panel shown specific ion capture from 449.05 to 449.20 m/z. Forth panel shows the strongest specific peaks from the full MS survey scan range of 200-2000 m/z at the retention time of 11.81-11.33 minutes.

Supplemental Figure S8

CitWax 9-9
MS2

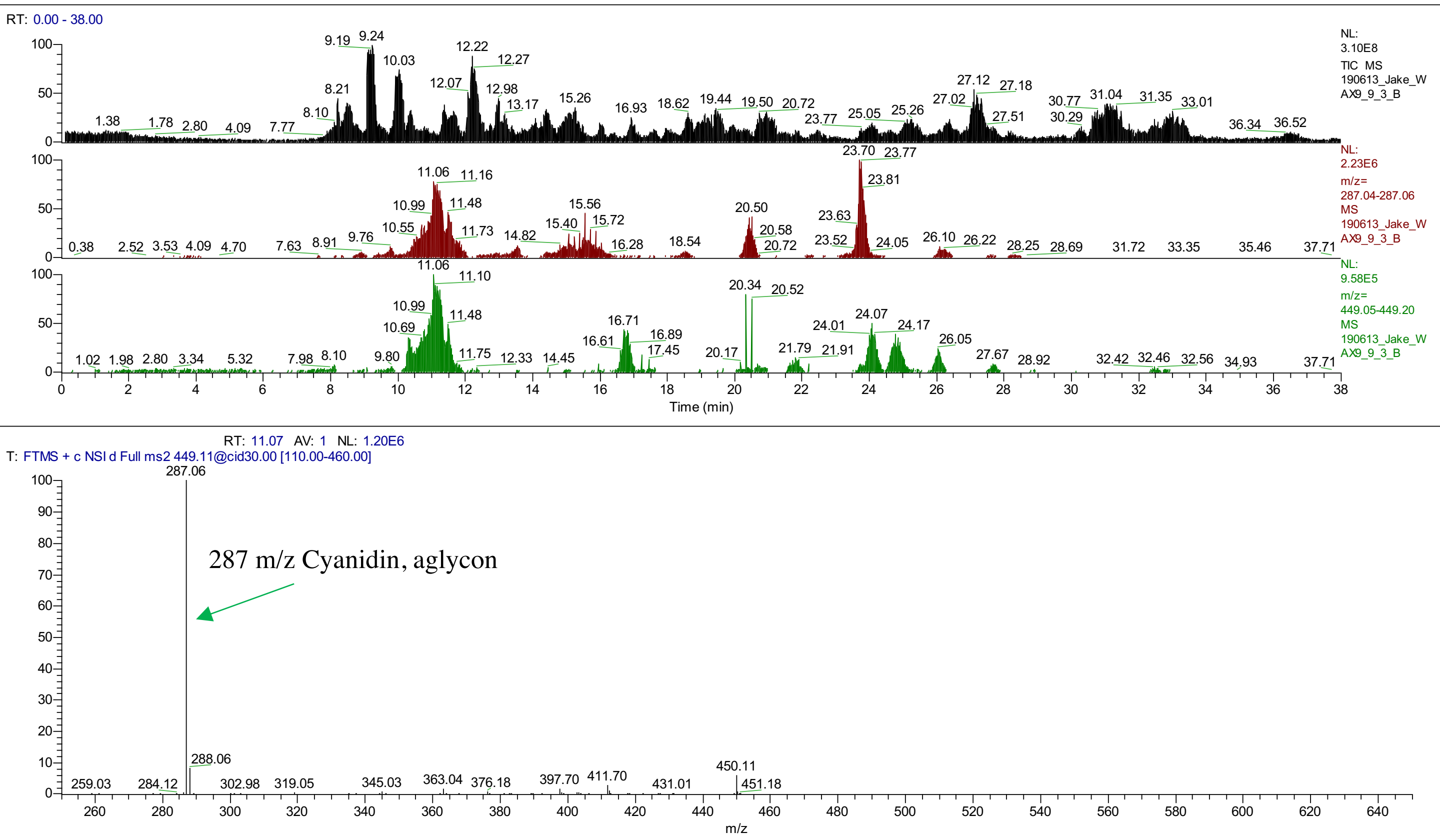

Supplemental Figure 8G. CitWax 9-9 juice MS2 analysis. Top panel shows the ion capture of all charge in compounds from MS survey scan range of 200-2000 m/z . Second panel shows specific ion capture from 287.04 to 287.06 m/z. Third panel shown specific ion capture from 449.05 to 449.20 m/z. Forth panel shows the strongest peaks after electrospray fragmentation from the full MS survey scan range of 449.11 m/z at the retention times 11.07 minutes.
